# Supplementary material for: DNA-PK deficiency potentiates cGAS-mediated antiviral innate immunity
Source: Nat Commun. 2020 Dec 3;11:6182. doi: 10.1038/s41467-020-19941-0 (PMC7712783; doi:10.1038/s41467-020-19941-0)
Supplement: Supplementary file 1 — Supplementary Information [file 41467_2020_19941_MOESM1_ESM.pdf]

Supplementary information

DNA-PK deficiency potentiates cGAS-mediated antiviral innate immunity

Sun et al.

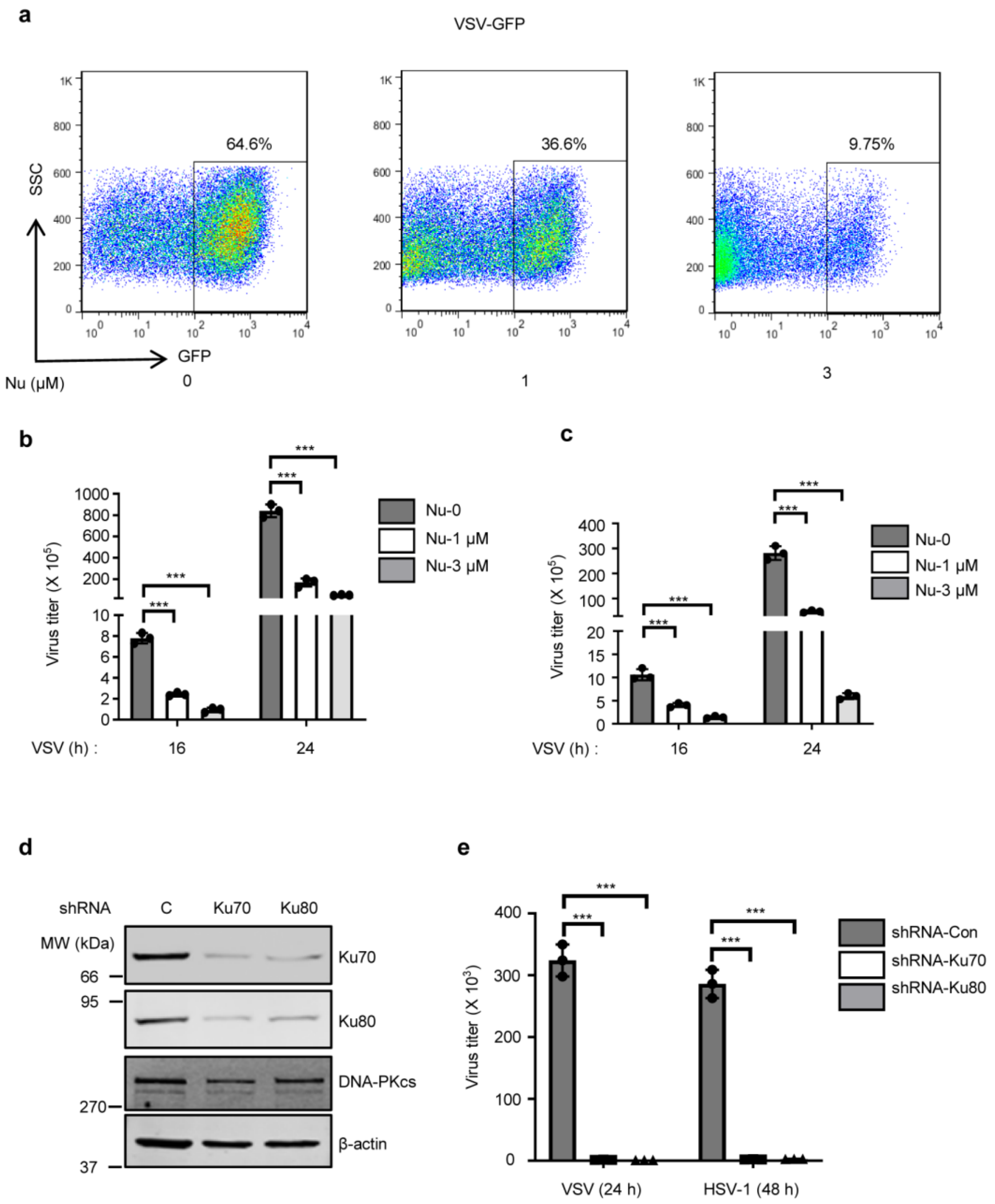

Supplementary Fig. 1. DNA-PK inhibition suppresses VSV and HSV-1 replication.

a, THP-1 cells were treated with Nu7441 and infected with VSV-GFP (MOI=0.01). GFP-positive cell percentage was quantified by Flow Cytometry at 16 h post-infection.

b-c, THP-1 or L929 cells were treated with Nu7441 and infected with VSV (MOI=0.01). Viral titer in the medium at the indicated time points was determined by plaque assay. **b.** VSV (16h): $p=6x10^{-5}$ (Nu-1 μM), $p=3x10^{-5}$ (Nu-3 μM); VSV (24h): $p=8x10^{-5}$ (Nu-1 μM), $p=2x10^{-5}$ (Nu-3 μM). **c.** VSV (16h): p=0.0009 (Nu-1 μM), p=0.0002 (Nu-3 μM); VSV (24h): p=0.0001 (Nu-1 μM), $p=6x10^{-5}$ (Nu-3 μM).

d, THP-1 cells infected with control (C), Ku70 or Ku80 shRNA lentivirus were selected with puromycin, and whole-cell lysates (WCLs) were analyzed by immunoblotting with the indicated antibodies.

e, THP-1 stable cells as described in (d) were infected with VSV or HSV-1 (MOI=0.01). Viral titer in the medium at the indicated time points were determined by plaque assays. VSV (24h): $p=3x10^{-5}$ (Ku70), $p=3x10^{-5}$ (Ku80); HSV-1 (48h): $p=3x10^{-5}$ (Nu-1 µM), $p=3x10^{-5}$ (Nu-3 µM).

All experiments were done at least twice, and one representative is shown. n=3 biologically independent samples for **b, c, e**. Data are presented as mean values +/- SD. ***p<0.005, two-tailed Student's t-test. Source data are provided as a Source Data file.

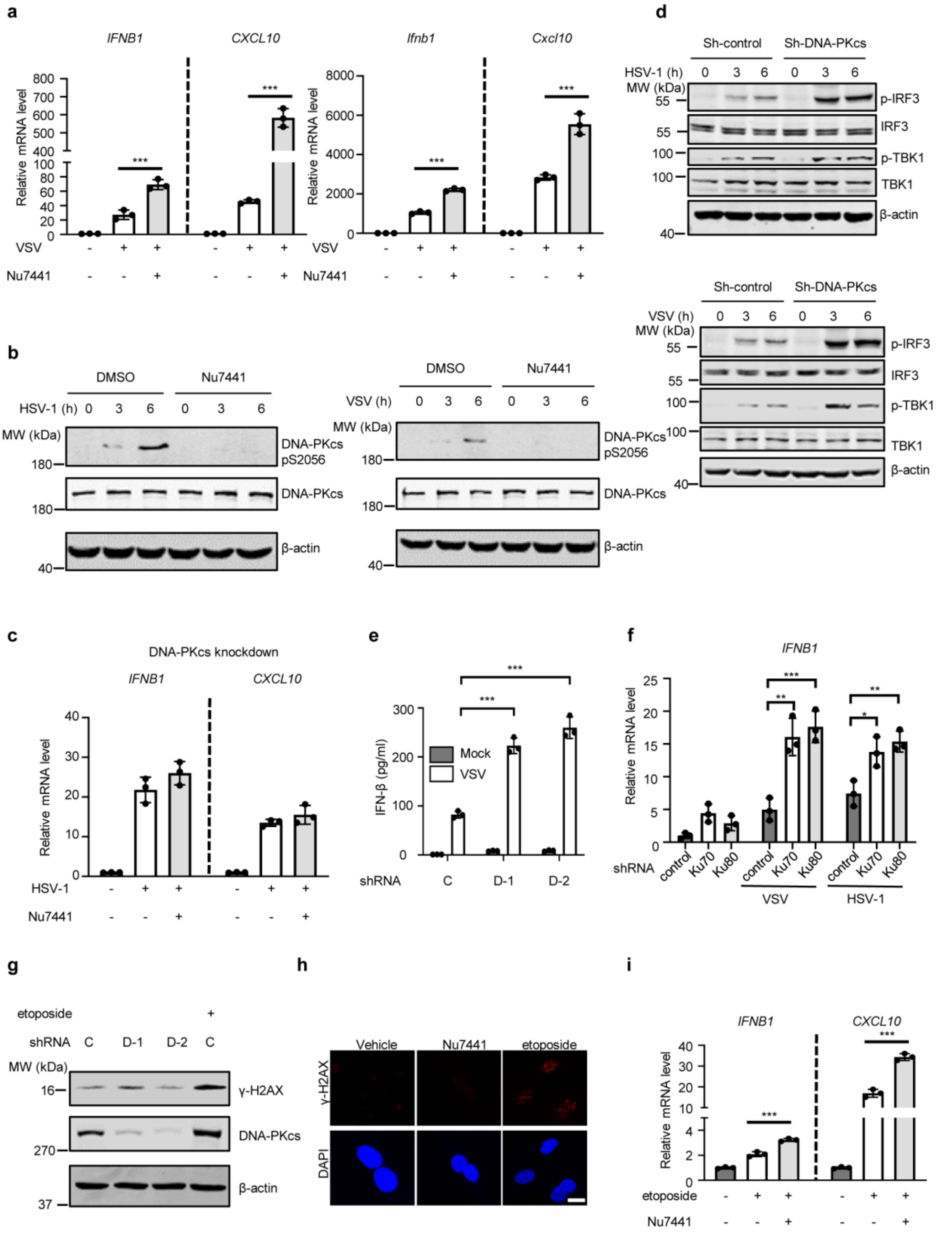

Supplementary Fig. 2. DNA-PK deficiency potentiates stronger innate immune responses.

a, THP-1 or L929 cells were treated with Nu7441 (3 μM) and infected with VSV (MOI=5). Infected cells were harvested at 6 hpi, and the expression of indicated cytokine genes was analyzed by real-time PCR. *IFNB1*: p=0.0016, *CXCL10*: p=5x10^{-5} (left panel); *Ifnb1*: p=3x10^{-5}, *Cxcl10*: p=0.0010 (right panel).

b, THP-1 cells were mock-treated or treated with Nu7441 (3 μM) and infected with HSV-1 or VSV (MOI=5). Cells were harvested at the indicated time points and whole cell lysates (WCLs) were analyzed by immunoblotting with the indicated antibodies.

c, THP-1 cells stably expressing DNA-PKcs shRNA were infected with HSV-1 (MOI=5) and treated with Nu7441 (3 μM). Cells were harvested at 6 hpi, and the expression of indicated cytokine genes was analyzed by real-time PCR.

d, THP-1 cells stably expressing control or DNA-PKcs shRNA were infected with HSV-1 or VSV (MOI=5). Cells were harvested at the indicated time points and WCLs were analyzed by immunoblotting with the indicated antibodies.

e, THP-1 cells stably expressing control or DNA-PKcs shRNA were infected with VSV (MOI=0.05). Infected cells were harvested at 16 hpi, and IFN-β was determined by ELISA. p=0.0002 (D-1), p=0.0002 (D-2).

f, THP-1 cells stably expressing control, Ku70 or Ku80 shRNA were infected with VSV (MOI=5). Infected cells were harvested at 6 hpi, and the expression of *IFNB1* was determined by real-time PCR. VSV: p=0.0054 (Ku70), p=0.0018 (Ku80); HSV-1: p=0.021 (Ku70), p=0.0055 (Ku80).

g, THP-1 cells stably expressing control or DNA-PKcs shRNA were mock-treated or treated with etoposide (10 μM) for 2 h. WCLs were analyzed by immunoblotting with the indicated antibodies.

h, THP-1 cells mock-treated or treated with Nu7441 (3 μM) for 6 h or etoposide (10 μM) for 2 h. γ-H2AX were detected by immunofluorescence. (Scale bar = 10 μm).

i, THP-1 cell were treated with Nu7441 (3 μM) and etoposide (10 μM) for 8 h, and the expression of *IFNB1* was determined by real-time PCR. *IFNB1*: p=0.0009, *CXCL10*: p=0.0003.

All experiments were done at least twice, and one representative is shown. n=3 biologically independent samples for **a, c, e, f, i**. Data are presented as mean values +/- SD. *p<0.05, **p<0.01, ***p<0.005, two-tailed Student's t-test. Source data are provided as a Source Data file.

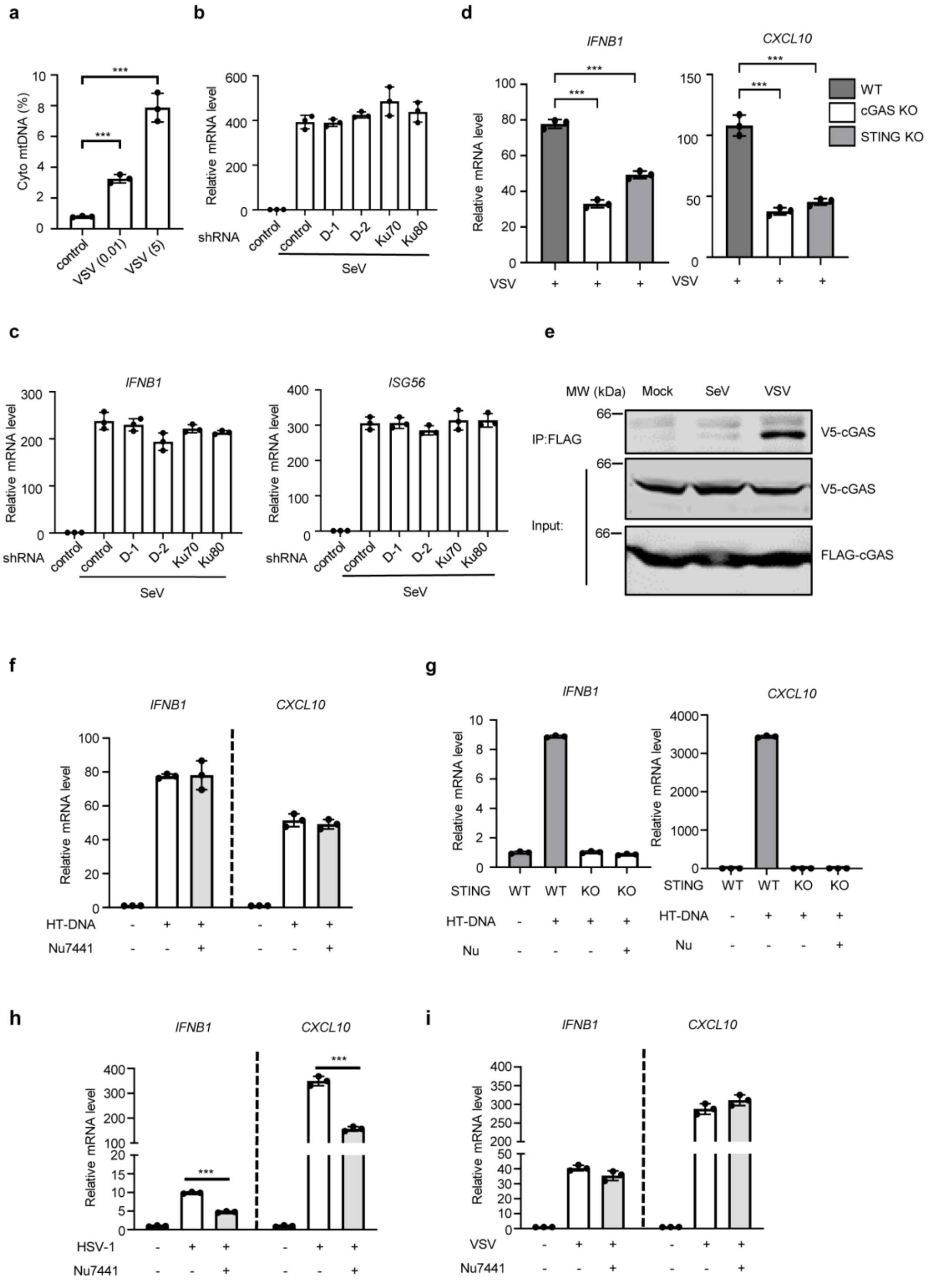

Supplementary Fig. 3. DNA-PK inhibition enhances cGAS-mediated antiviral immune responses.

a, THP-1 cells were infected with VSV (MOI=0.01 or 5). Cytoplasmic mitochondrial DNA was extracted and quantified by real-time PCR at 6 hpi. p=0.0031 (VSV-0.01); p=0.0002 (VSV-5).

b-c, THP-1 cells stably expressing control, DNA-PKcs, Ku70 or Ku80 shRNA were infected with SeV (20 HAU/ml). Infected cells were harvested at 6 hpi, and the expression of viral and indicated cytokine genes was analyzed by real-time PCR.

d, THP-1 cells as described in Fig. 3a were infected with VSV (MOI=5). Infected cells were harvested at 6 hpi, and the expression of viral and indicated cytokine genes was analyzed by real-time PCR. *IFNB1*: p=2x10^{-5} (cGAS KO), p=0.0001 (STING KO); *CXCL10*: p=0.0002 (cGAS KO), p=0.0003 (STING KO).

e, 293T cells stably expressing FLAG-cGAS and V5-cGAS were mock-infected or infected with SeV (20 HAU/ml) or VSV (MOI=0.01) for 16 h. WCLs were precipitated with anti-FLAG agarose. Precipitated proteins and WCLs were analyzed by immunoblotting with the indicated antibodies.

f, THP-1 cells stably expressing DNA-PKcs shRNA were transfected with HT-DNA (1 µg/ml) and treated with Nu7441 (3 µM) for 6 h. The expression of indicated cytokine genes was analyzed by real-time PCR.

g, THP-1 or THP-1 STING KO cells were transfected with HT-DNA (1 µg/ml) for 6 h, and the expression of indicated cytokine genes was analyzed by real-time PCR.

h-i, THP-1 STING KO cells were infected with HSV-1 or VSV (MOI=5) and treated with Nu7441 (3 µM). Infected cells were harvested at 6 hpi, and the expression of indicated cytokine genes was analyzed by real-time PCR. **h.** *IFNB1*: p=7x10^{-6}, *CXCL10*: p=0.0001.

All experiments were done at least twice, and one representative is shown. n=3 biologically independent samples for **a-d, f-i**. Data are presented as mean values +/- SD. ***p<0.005, two-tailed Student's t-test. Source data are provided as a Source Data file.

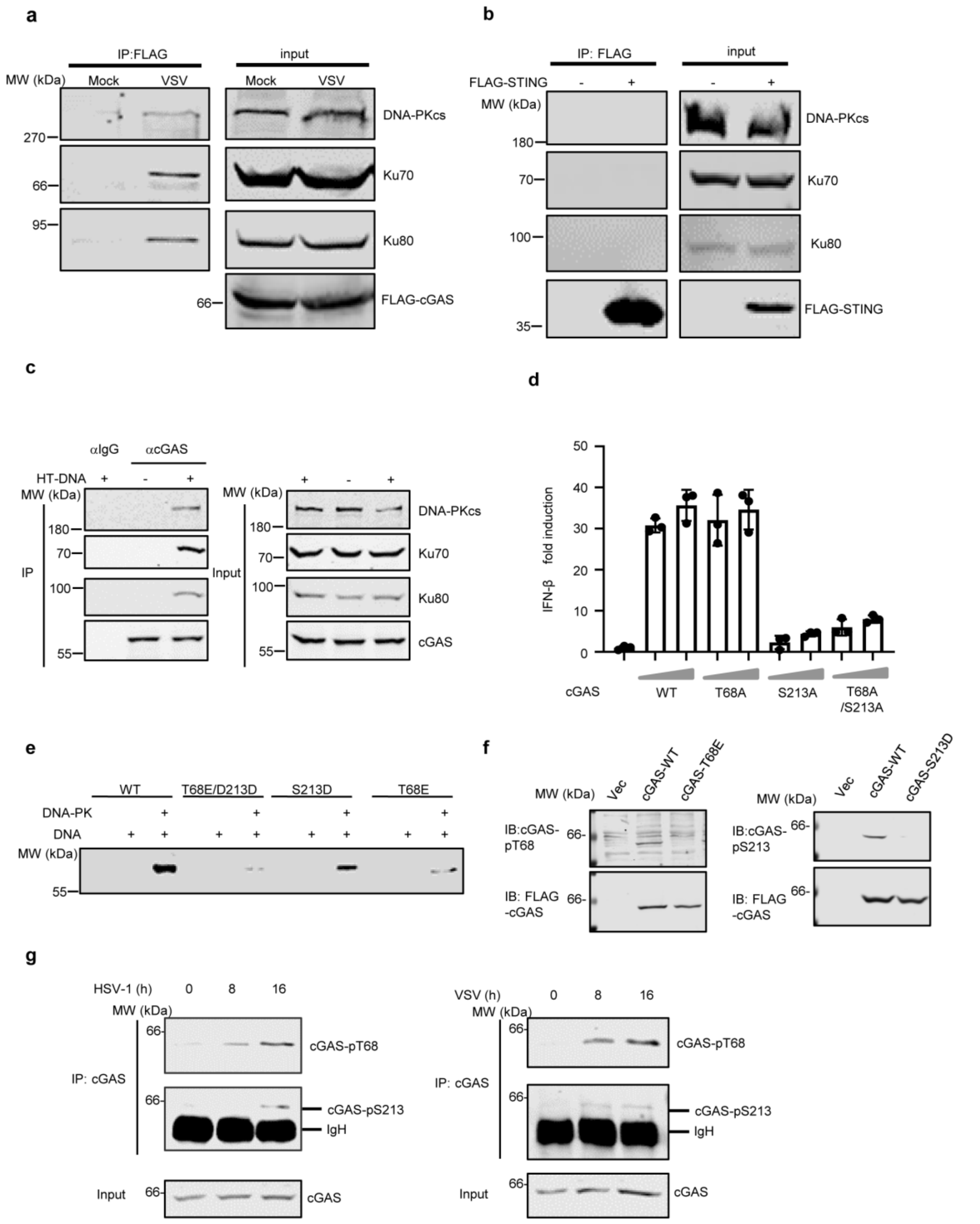

Supplementary Fig. 4. DNA-PK phosphorylates cGAS.

a, THP-1 cells stably expressing FLAG-cGAS were mock-infected or infected with VSV (MOI=0.01) for 16 h. WCLs were precipitated with anti-FLAG agarose. Precipitated proteins and WCLs were analyzed by immunoblotting with the indicated antibodies.

b, THP-1 cells stably expressing vector or FLAG-STING were infected with VSV (MOI=0.01) for 16 h. WCLs were precipitated with anti-FLAG agarose. Precipitated proteins and WCLs were analyzed by immunoblotting with the indicated antibodies.

c, THP-1 cells were transfected with HT-DNA (1 μg/ml) for 6 h before co-immunoprecipitation

and immunoblotting analysis.

d, 293T cells were transfected with an IFN-β reporter plasmid cocktail with plasmids containing cGAS WT or the mutants and STING. IFN-β activation was determined by luciferase assay.

e, *In vitro* kinase assay was performed using DNA-PK complex and purified cGAS or the mutants with HT-DNA (1 μg/ml).

f, 293T cells were transfected with cGAS WT or the mutants and WCLs were analyzed by immunoblotting with the indicated antibodies.

g, THP-1 cells were mock-infected or infected with HSV-1 (MOI=1) or VSV (MOI=0.1) for 8 h and 16 h before co-immunoprecipitation and immunoblotting analysis.

All experiments were done at least twice, and one representative is shown. n=3 biologically independent samples for **d**. Data are presented as mean values +/- SD. Source data are provided as a Source Data file.

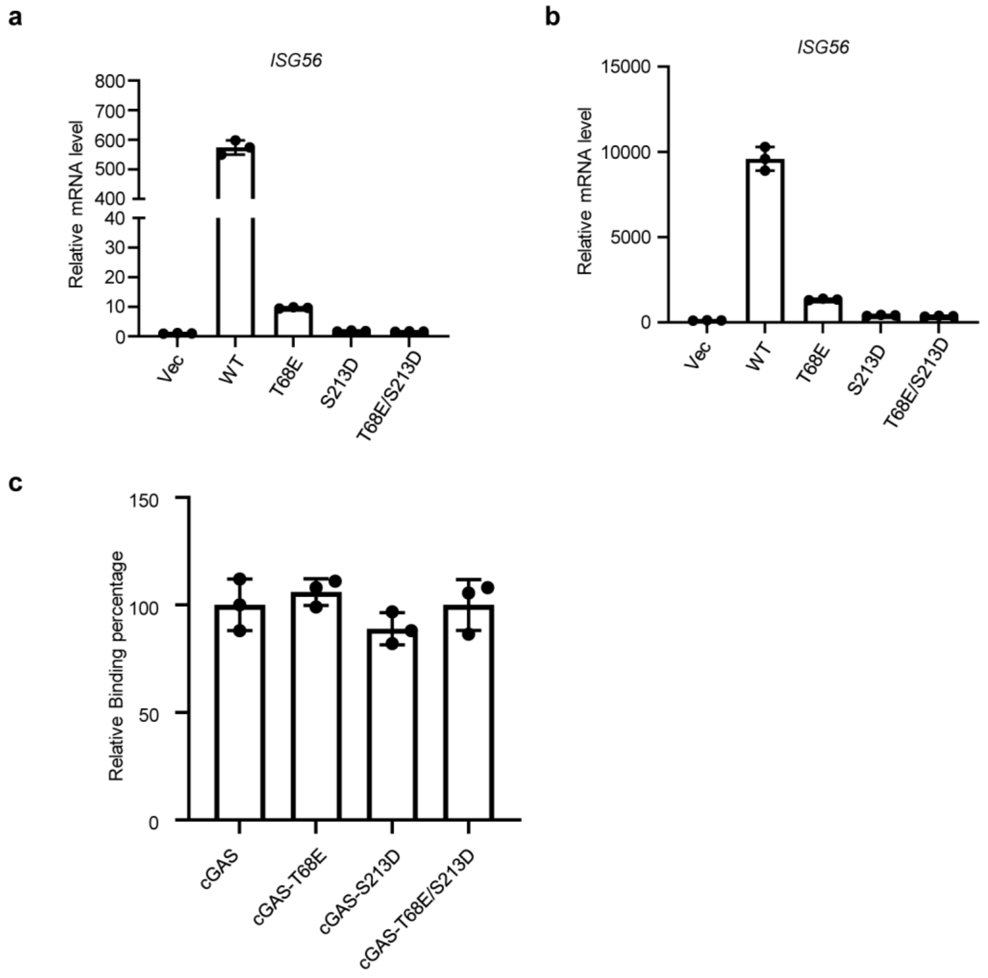

Supplementary Fig. 5. cGAS phosphorylation by DNA-PK inhibits the activity of cGAS.

a, The expression of *Isg56* in reconstituted cGAS stable cells was analyzed by real-time PCR.

b, Reconstituted cGAS stable cells were stimulated with HT-DNA (1 μg/mL). Cells were harvested at 6 h post transfection, and the expression of *Isg56* was analyzed by real-time PCR.

c, WCLs were prepared from 293T cells transiently transfecting cGAS WT or the mutants. Biotinylated interferon-stimulating DNA (Biotin-ISD) was used to pull down cGAS WT or the mutants *in vitro*, and precipitated proteins were analyzed by immunoblotting and binding percentage was quantified.

All experiments were done at least twice, and one representative is shown. n=3 biologically independent samples for **a-c**. Data are presented as mean values +/- SD. Source data are provided as a Source Data file.

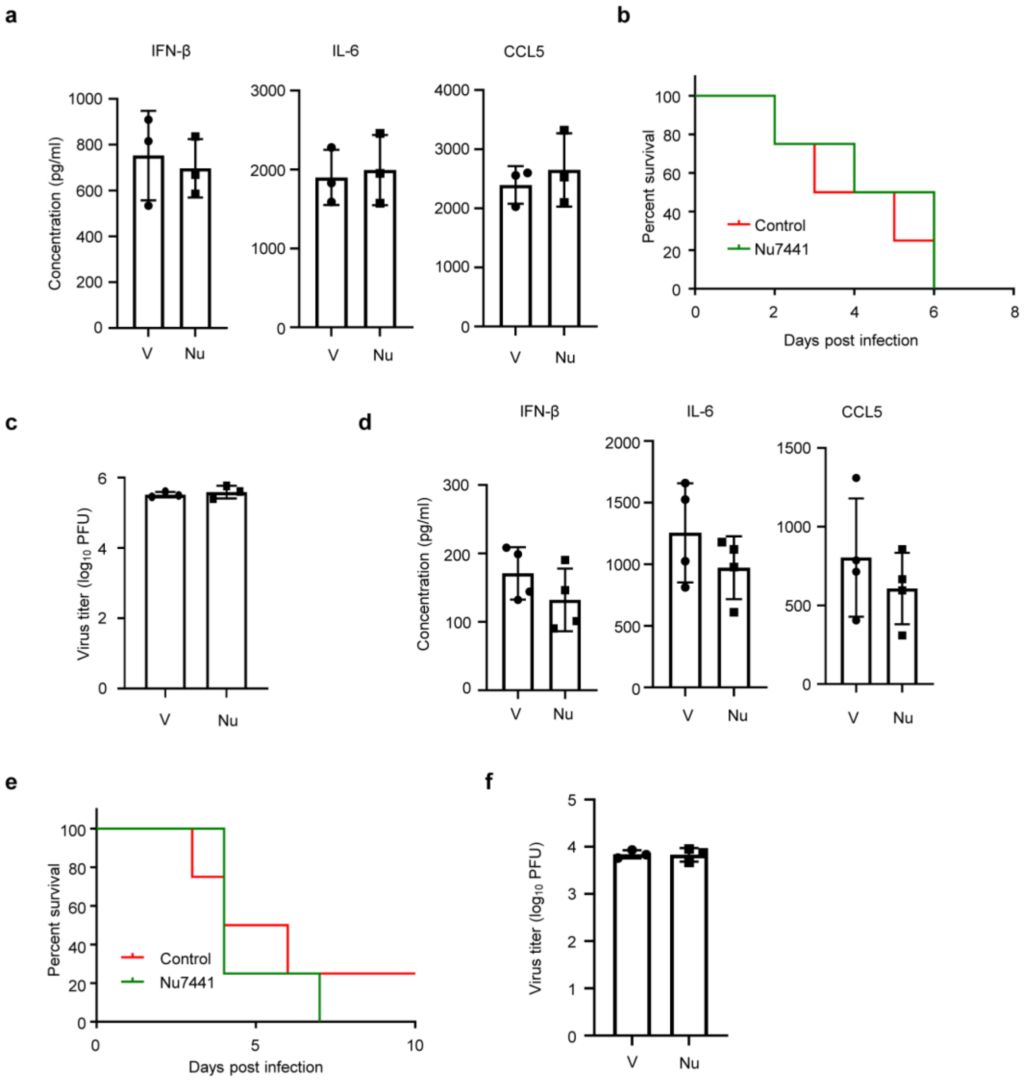

Supplementary Fig. 6. Nu7441 treatment promotes antiviral innate immunity and suppresses VSV and HSV-1 replication *in vivo*.

a-c, Age- and gender-matched cGAS KO mice were treated with 20mg/kg Nu7441 or control through intraperitoneal injection and infected with VSV (10^8 PFU). (a) Blood was collected at 6 h post-infection, and indicated cytokines in sera were determined by ELISA (n=3 biologically independent animals). (b) Mouse survival was recorded and shown as percentage over time (n=4 biologically independent animals). (c) Mice were sacrificed at 3

days post-infection, and viral titers in the spleens were quantified by plaque assays (n=3 biologically independent animals).

d-f, Age- and gender-matched cGAS KO mice were treated with 20mg/kg Nu7441 or control through intraperitoneal injection and infected with HSV-1 ($5x10^7$ PFU). (d) Blood was collected at 6 h post-infection, and indicated cytokines in sera were determined by ELISA (n=4 biologically independent animals). (e) Mouse survival was recorded and shown as percentage over time (n=4 biologically independent animals). (f) Mice were sacrificed at 3 days post-infection, and viral titers in the brains were quantified by plaque assays (n=3 biologically independent animals).

All experiments were done at least twice, and one representative is shown. For **a, c, d, f,** data are presented as mean values +/- SD. Two-tailed Student's t-test (a, c, d, f) and log-rank test (b, e). Source data are provided as a Source Data file.

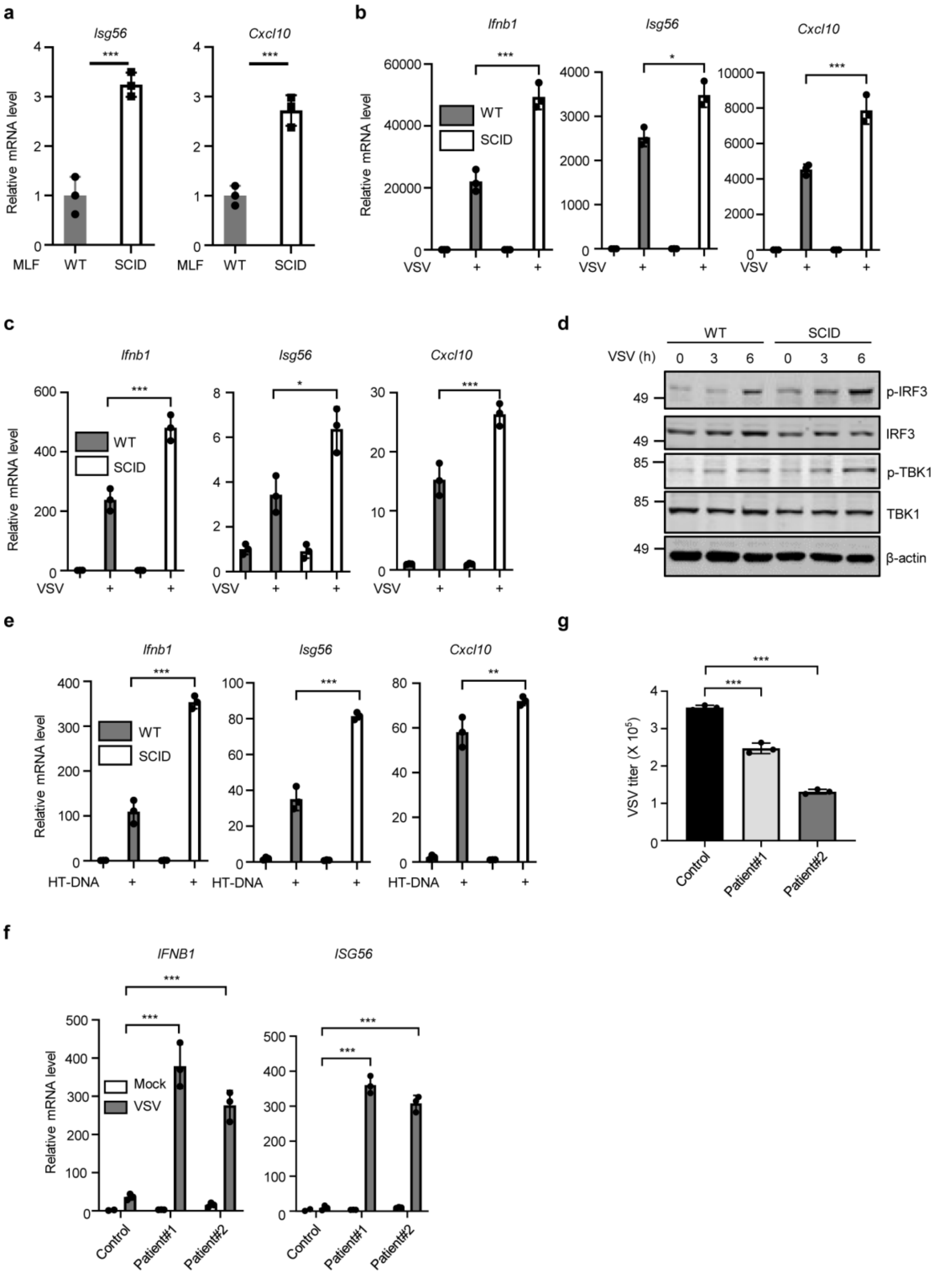

Supplementary Fig. 7. DNA-PK deficiency potentiates antiviral innate immunity in cells isolated from mice and patients.

a, RNA was extracted from MLFs generated from WT or SCID mice and the expression of the indicated genes was analyzed by real-time PCR. p=0.0010 (*Isg56*); p=0.0012 (*Cxcl10*).

b, MLFs were infected with VSV (MOI=5). Cells were harvested at 6 h post-infection, and the expression of the indicated genes was analyzed by real-time PCR. p=0.0009 (*Ifnb1*); p=0.028(*Isg56*); p=0.0023 (*Cxcl10*).

c, Bone marrow-derived macrophages (BMDMs) were infected with VSV (MOI=5). Total RNA was extracted at 6 h post-infection and the expression of the indicated genes was determined by real-time PCR. p=0.0019 (*Ifnb1*); p=0.016(*Isg56*); p=0.0046 (*Cxcl10*).

d, MLFs generated from WT or SCID mice were infected with VSV (MOI=5). Cells were harvested at the indicated time points, and WCLs were analyzed by immunoblotting with the indicated antibodies.

e, BMDMs generated from WT or SCID mice were transfected with HT-DNA (1 μg/mL). Cells were harvested at 6 h post-transfection, and the expression of the indicated genes was analyzed by real-time PCR. p=0.0001 (*Ifnb1*); p=0.0003 (*Isg56*); p=0.026 (*Cxcl10*).

f, Fibroblasts generated from control subject or patients with DNA-PKcs mutations were infected with VSV (MOI=5). Cells were harvested at 6 h post-transfection, and the expression of the indicated genes was analyzed by real-time PCR. *IFNB1*: p=0.0005 (patient#1), p=0.0005 (patient#2); *ISG56*: p=$2x10^{-5}$ (patient#1), p=$3x10^{-5}$ (patient#2);

g, Fibroblasts generated from control subject or patients with DNA-PKcs mutations were infected with VSV (MOI=0.05). Viral titer in the medium was determined by plaque assays at 24 h post-infection. p=0.0002 (patient#1), p=$2x10^{-5}$ (patient#2).

All experiments were done at least twice, and one representative is shown. n=3 biologically independent samples for **a-c, e-g**. Data are presented as mean values +/- SD. *p<0.05, **p<0.01, ***p<0.005, two-tailed Student's t-test. Source data are provided as a Source Data file.

Supplementary Table

Q-PCR primers for human genes:		
IFNB1	CAGGAGAGCAATTTGGAGGA	CTTTCGAAGCCTTTGCTCTG
ISG56	TCTCAGAGGAGCCTGGCTAA	TGACATCTCAATTGCTCCAG
CXCL10	CACCATGAATCAAACTGCGA	GCTGATGCAGGTACAGCGT
ACTB<br>(β-actin)	GTTGTCGACGACGAGCG	GCACAGAGCCTCGCCTT
Q-PCR primers for mouse genes:		
Ifnb1	CCCTATGGAGATGACGGAGA	CCCAGTGCTGGAGAAATTGT
Isg56	CAAGGCAGGTTTCTGAGGAG	GACCTGGTCACCATCAGCAT
Cxcl10	CTCATCCTGCTGGGTCTGAG	CCTATGGCCCTCATTCTCAC
Actb<br>(β-actin)	TCTACGAGGGCTATGCTCTCC	TCTTTGATGTCACGCACGATTTC
Primers for quantification of mitochondrial DNA		
EGFP	ACGGCGACGTAAACGGCCAC	GCACGCCGTAGGTCAGGGTG
mtDNA1	CACCCAAGAACAGGGTTTGT	TGGCCATGGGTATGTTGTTAA
mtDNA2	CTATCACCCTATTAACCACTCA	TTCGCCTGTAATATTGAACGTA
