# Supplementary material for: DNA-PK deficiency potentiates cGAS-mediated antiviral innate immunity
Source: Nat Commun. 2020 Dec 3;11:6182. doi: 10.1038/s41467-020-19941-0 (PMC7712783; doi:10.1038/s41467-020-19941-0)
Supplement: Supplementary file 3 — Reporting Summary [file 41467_2020_19941_MOESM3_ESM.pdf]

nature research

Corresponding author(s): Junjie Zhang

Last updated by author(s): 2020-11-5

Reporting Summary

Nature Research wishes to improve the reproducibility of the work that we publish. This form provides structure for consistency and transparency in reporting. For further information on Nature Research policies, see our Editorial Policies and the Editorial Policy Checklist.

Statistics

For all statistical analyses, confirm that the following items are present in the figure legend, table legend, main text, or Methods section.

n/a	Confirmed	
☐	☒	The exact sample size (*n*) for each experimental group/condition, given as a discrete number and unit of measurement
☐	☒	A statement on whether measurements were taken from distinct samples or whether the same sample was measured repeatedly
☐	☒	The statistical test(s) used AND whether they are one- or two-sided<br>*Only common tests should be described solely by name; describe more complex techniques in the Methods section.*
☒	☐	A description of all covariates tested
☒	☐	A description of any assumptions or corrections, such as tests of normality and adjustment for multiple comparisons
☐	☒	A full description of the statistical parameters including central tendency (e.g. means) or other basic estimates (e.g. regression coefficient) AND variation (e.g. standard deviation) or associated estimates of uncertainty (e.g. confidence intervals)
☐	☒	For null hypothesis testing, the test statistic (e.g. *F*, *t*, *r*) with confidence intervals, effect sizes, degrees of freedom and *P* value noted<br>*Give P values as exact values whenever suitable.*
☒	☐	For Bayesian analysis, information on the choice of priors and Markov chain Monte Carlo settings
☒	☐	For hierarchical and complex designs, identification of the appropriate level for tests and full reporting of outcomes
☒	☐	Estimates of effect sizes (e.g. Cohen's *d*, Pearson's *r*), indicating how they were calculated

Our web collection on statistics for biologists contains articles on many of the points above.

Software and code

Policy information about availability of computer code

Data collection	Bio-Rad CFX manager for qPCR, NIS-Elements for confocal, BMG plate-reader for ELISA, BD FACS Canto II for flow cytometry and Licor Odyssey 9120 for Western Blot, SEQUEST 1.0 for mass spectrometry.
Data analysis	Graphpad Prism 7.0 for statistical analysis, FlowJo 7.6 for FACS data analysis.

For manuscripts utilizing custom algorithms or software that are central to the research but not yet described in published literature, software must be made available to editors and reviewers. We strongly encourage code deposition in a community repository (e.g. GitHub). See the Nature Research guidelines for submitting code & software for further information.

Data

Policy information about availability of data

All manuscripts must include a data availability statement. This statement should provide the following information, where applicable:

- Accession codes, unique identifiers, or web links for publicly available datasets
- A list of figures that have associated raw data
- A description of any restrictions on data availability

All data are included in the Supplemental Information or available from the authors upon reasonable requests. Source data are provided with this paper.

Field-specific reporting

Life sciences study design

All studies must disclose on these points even when the disclosure is negative.

Sample size	No statistical methods were used to predetermine sample size. Sample size was based on empirical data from pilot experiments. The detail was Reported in the Statistical Analysis section.
Data exclusions	No data were excluded from the studies
Replication	Experiments were repeated at least twice to ensure reproducibility of data as indicated in the Figure Legends. All attempts are successful.
Randomization	For mouse infection experiments, C57BL/6 WT or cGAS KO mice were divided randomly into cages. During experiments, mice with the same sex (irrespective of whether they are treated with vehicle control or the compound) were housed in the same cage to avoid any bias due to location.
Blinding	For in vivo infection experiments the researchers were not blinded since they need to treat the mice first and then perform the infection experiments afterwards. However, at least two to three researchers performed the experiments and collected the samples at the same time to exclude the bias. For experiments other than mouse infection, the researchers were not blinded during data collection and analysis. However, the experiments are repeated by independent researchers to validate the results.

Reporting for specific materials, systems and methods

We require information from authors about some types of materials, experimental systems and methods used in many studies. Here, indicate whether each material, system or method listed is relevant to your study. If you are not sure if a list item applies to your research, read the appropriate section before selecting a response.

Materials & experimental systems

n/a	Involved in the study	
☐	☒	Antibodies
☐	☒	Eukaryotic cell lines
☒	☐	Palaeontology and archaeology
☐	☒	Animals and other organisms
☐	☒	Human research participants
☒	☐	Clinical data
☒	☐	Dual use research of concern

Methods

n/a	Involved in the study	
☒	☐	ChIP-seq
☐	☒	Flow cytometry
☒	☐	MRI-based neuroimaging

Antibodies

Antibodies used	Commercially available antibodies used for this study include mouse monoclonal FLAG M2 antibody (Sigma); rabbit polyclonal V5 antibody (Thermo Fisher); mouse monoclonal V5, b-actin and TBK1 antibody (Abcam); Phospho-TBK1 (Ser172), Phospho-IRF3 (Ser396), Ku80, cGAS (D1D3G) and STING (D2P2F) antibody (Cell signaling); GAPDH (6C5), VDAC1 (B-6) and IRF3 antibody (FL-425) (Santa Cruz); DNA-PKcs and Ku70 antibody (NeoMarkers); phospho-Histone H2A.X (Ser139)/γ-H2AX Antibody (Millipore).<br>Rabbit polyclonal anti-cGAS pT68 and pS213 antibodies were generated by repeated immunization with the phospho-specific peptides (GenScript). Rabbit polyclonal anti-MAVS antibody was home-made in the lab.
Validation	Many antibodies are already validated based on manufacturer's data and they are widely used in various published articles:<br>FLAG M2 antibody (Sigma) (PMID: 26221961); rabbit polyclonal V5 antibody (Thermo Fisher) (PMID: 30092200); mouse monoclonal V5 (PMID: 30092200), b-actin (PMID: 30092200) and TBK1 antibody (PMID: 30092200); Phospho-TBK1 (Ser172) (PMID: 30092200), Phospho-IRF3 (Ser396) (PMID: 30092200), Ku80 (PMID: 22542840), cGAS (D1D3G) (PMID: 30092200) and STING (D2P2F) antibody (PMID: 29653696)(Cell signaling); GAPDH (6C5) (PMID: 33116119), VDAC1 (B-6) (PMID: 30077079) and IRF3 antibody (FL-425) (PMID: 30092200)(Santa Cruz); DNA-PKcs (PMID: 12231622) and Ku70 (PMID: 10547363) antibody (NeoMarkers); phospho-Histone H2A.X (Ser139)/γ-H2AX Antibody (PMID: 22542840) (Millipore).<br>Rabbit polyclonal anti-cGAS pT68 and pS213 antibodies were validated by ourselves as indicated in the figure legends. Rabbit polyclonal anti-MAVS antibody was described and validated previously (PMID: 26221961).

Eukaryotic cell lines

Policy information about cell lines

Cell line source(s)	HEK293T, Vero and THP-1 cell lines were obtained from American Type Culture Collection center (ATCC). L929 WT and cGAS KO cells were provided by Dr. James Chen (UT Southwestern Medical Center) (PMID: 23258413). HFF cells were provided by Dr. Bo Zhong, (Wuhan University) and Dr. Min-Hua Luo (Wuhan Institute of Virology) (PMID: 30943264). Mouse bone marrow-derived macrophages (BMDM) and mouse lung fibroblasts (MLF) were generated as described in the methods section.
Authentication	HEK293T, Vero and THP-1Cell lines were purchased from genuine vendors (ATCC) and we did not authenticate them. We did

	not authenticate L929 cell lines, BMDM and MLF.
Mycoplasma contamination	All the cells were routinely tested for contamination by mycoplasma and tested negative cells were used in the study.
Commonly misidentified lines (See ICLAC register)	No commonly misidentified cell lines were used in this study.

Animals and other organisms

Policy information about studies involving animals; ARRIVE guidelines recommended for reporting animal research

Laboratory animals	Six to eight-week old, gender-matched mice were used for all experiments. Both male and female mice of all strain are used and the gender is not a consideration of the study. All mice were housed in a standard pathogen-free animal facility. BALB/c and BALB/c SCID mice were purchased from Hunan Silaikejingda Laboratory Animal Technology Co. Ltd (Changsha, China). C57BL/6J mice were purchased from the Jackson Laboratory. cGAS KO mice were provided by Dr. Hong-Bing Shu (Wuhan University).
Wild animals	No wild animals were used for this study.
Field-collected samples	This study did not involve samples collected from field.
Ethics oversight	The protocol was approved by the Institutional Animal Care and Use Committee (IACUC) of Medical Research Institute, Wuhan University.

Note that full information on the approval of the study protocol must also be provided in the manuscript.

Human research participants

Policy information about studies involving human research participants

Population characteristics	Patient fibroblasts with DNA-PKcs mutations were provided by Drs. Alexandre Belot (CIRI, International Center for Infectiology Research, Universite de Lyon) and Isbelle Rouvet (the Biotechnology Department, Hospices Civils de Lyon). Sanger sequencing identified a c.9185T>G (p.Leu3062Arg) homozygous missense mutation and a homozygous c.6340delGAG (p.Gly2113del) deletion in PRKDC in patient #1. An independent patient #2 has the same two homozygous mutations. The detailed clinical features of these two patients were described previously (PMID: 25842288).
Recruitment	All patients provided written informed consent for inclusion in the study. Samples of anonymous healthy controls were purchased from core facilities. We collect two indecent patient samples and this can largely exclude the potential biases involved in sample collections.
Ethics oversight	The study was approved by the Medical Ethics Committee of Medical Research Institute, Wuhan University (Wuhan, China) and was carried out in accordance with the Declaration of Helsinki principles.

Note that full information on the approval of the study protocol must also be provided in the manuscript.

Flow Cytometry

Plots

Confirm that:

- [x] The axis labels state the marker and fluorochrome used (e.g. CD4-FITC).
- [x] The axis scales are clearly visible. Include numbers along axes only for bottom left plot of group (a 'group' is an analysis of identical markers).
- [x] All plots are contour plots with outliers or pseudocolor plots.
- [x] A numerical value for number of cells or percentage (with statistics) is provided.

Methodology

Sample preparation	THP-1 cells were infected with VSV-GFP (MOI=0.01). The cells were fixed with 10% formaldehyde for 10 min at room temperature and GFP-positive cell percentage was quantified at 16 h post-infection.
Instrument	BD FACS Canto II
Software	FlowJo 7.6 for data analysis
Cell population abundance	n/a
Gating strategy	Forward versus side scatter (FSC vs SSC) gating was used to identify cells of interests and exclude debris. A forward scatter height (FSC-H) vs forward scatter area (FSC-A) was used to exclude doublets. For GFP positive measurement, we used non-infected cells as negative controls.

- [ ] Tick this box to confirm that a figure exemplifying the gating strategy is provided in the Supplementary Information.
