# Supplementary material for: DNA-PK deficiency potentiates cGAS-mediated antiviral innate immunity
Source: Nat Commun. 2020 Dec 3;11:6182. doi: 10.1038/s41467-020-19941-0 (PMC7712783; doi:10.1038/s41467-020-19941-0)

Figure 1e

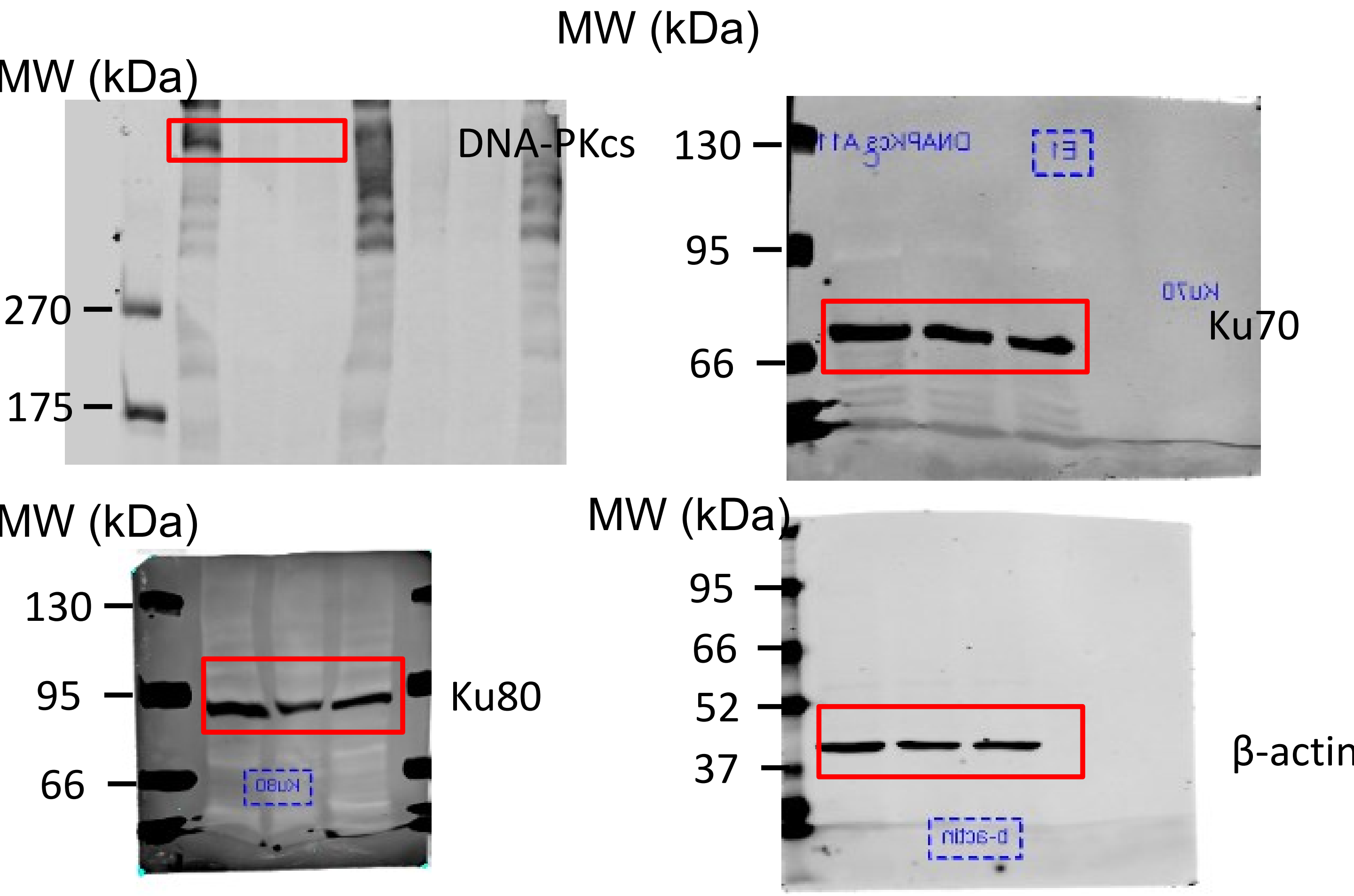

Figure S1d

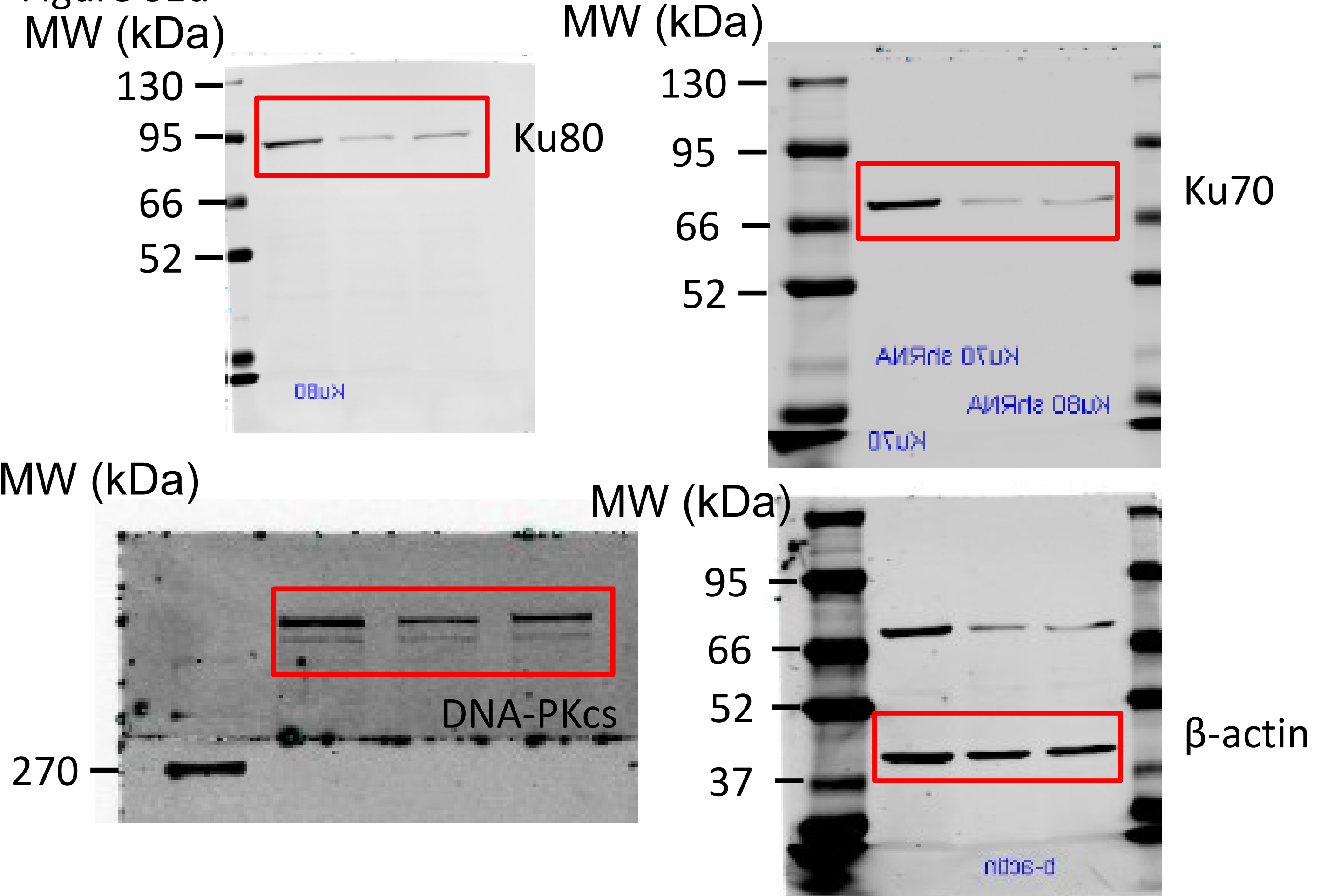

Figure 2c

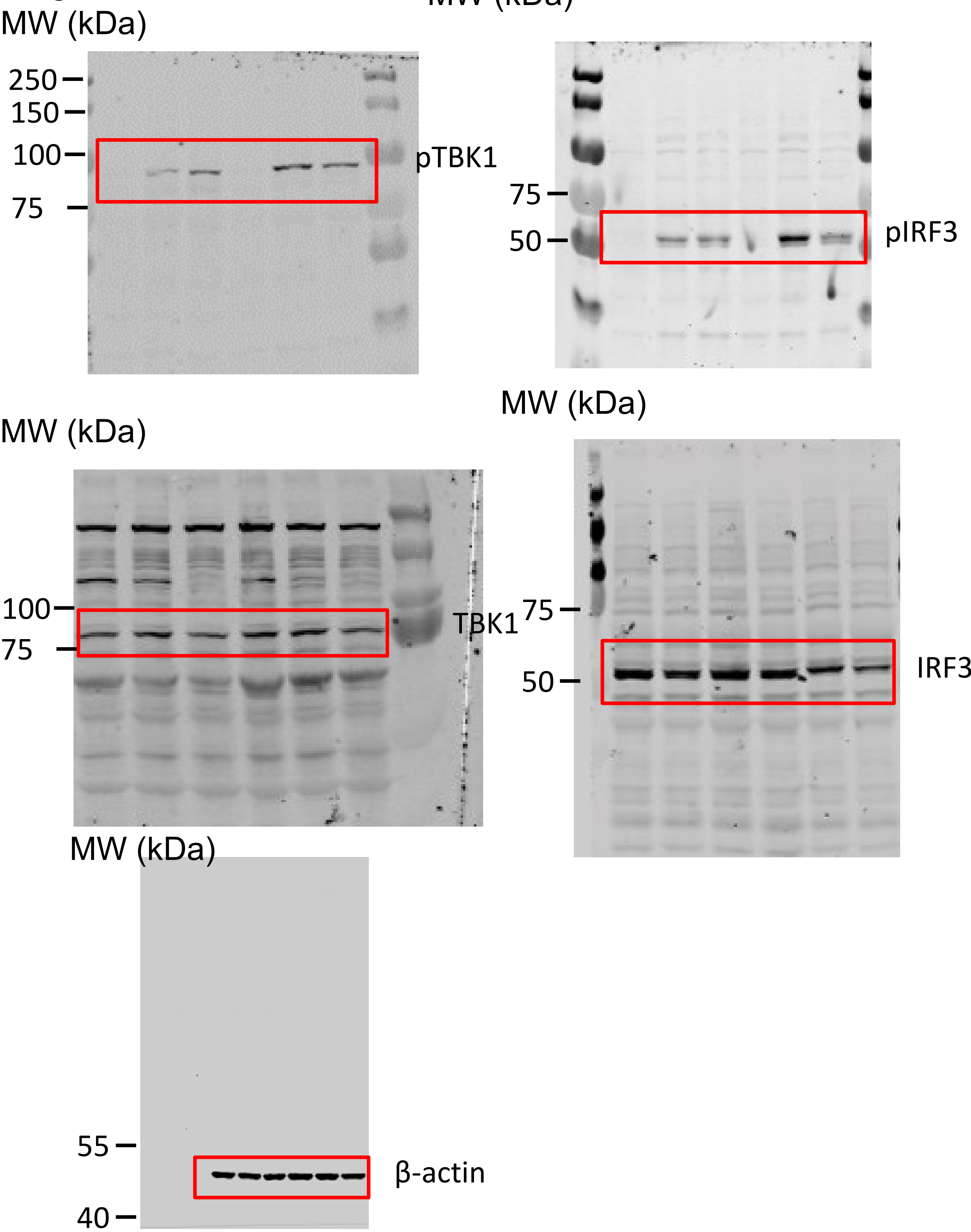

MW (kDa)
250
150
100
75
pTBK1
MW (kDa)
75
50
pIRF3
MW (kDa)
100
75
TBK1
MW (kDa)
75
50
IRF3
MW (kDa)
55
40
β-actin

Figure 2d

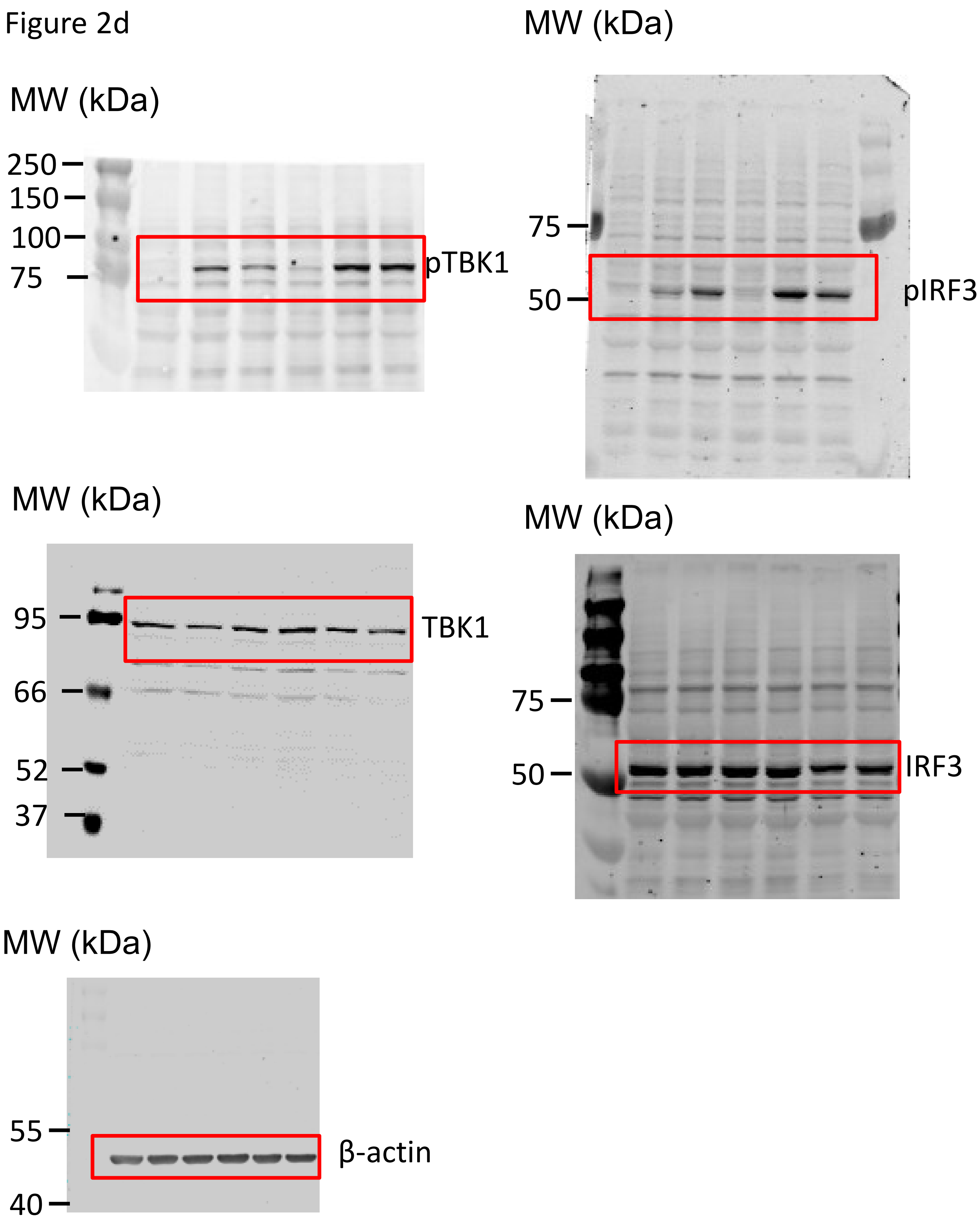

MW (kDa)
250
150
100
75
pTBK1
MW (kDa)
75
50
pIRF3
MW (kDa)
95
66
52
37
TBK1
MW (kDa)
75
50
IRF3
MW (kDa)
55
40
β-actin

Figure S2b

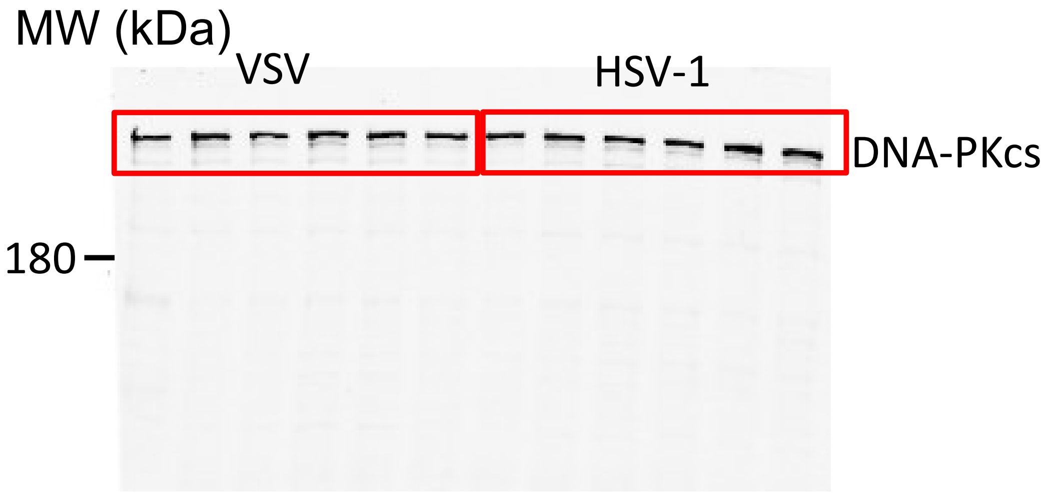
MW (kDa)
VSV
HSV-1
DNA-PKcs
180

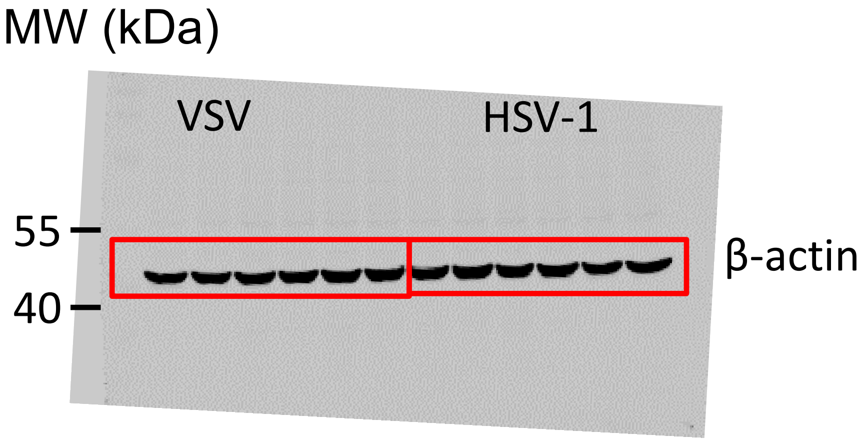
MW (kDa)
VSV
HSV-1
55
β-actin
40

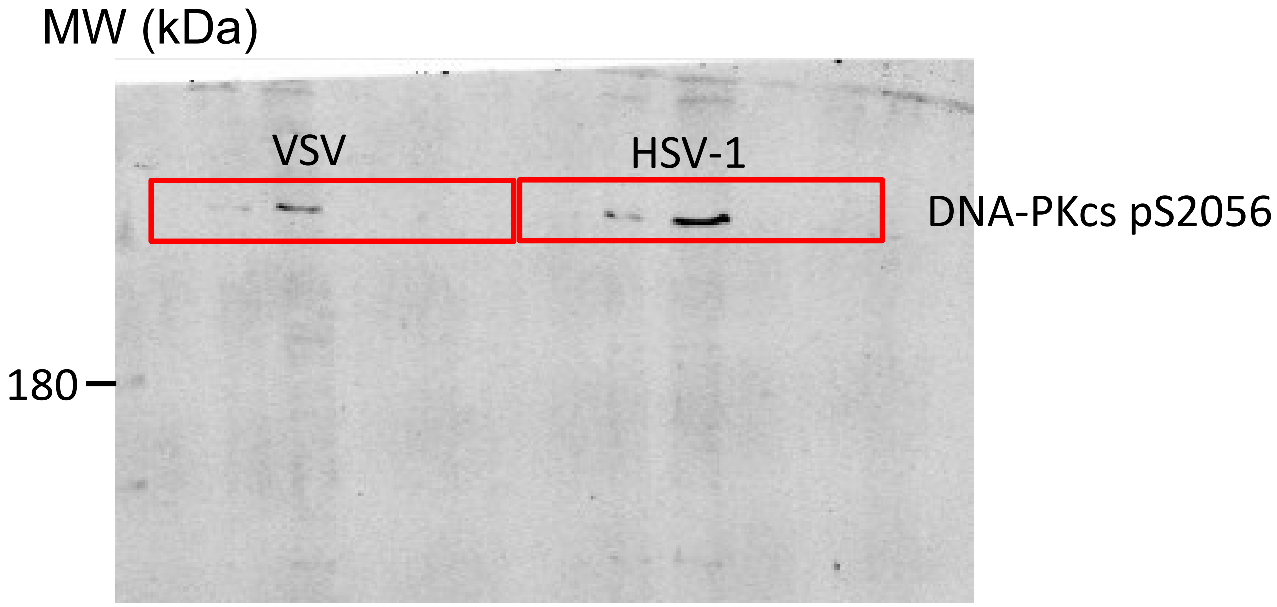
MW (kDa)
VSV
HSV-1
DNA-PKcs pS2056
180

Figure S2d

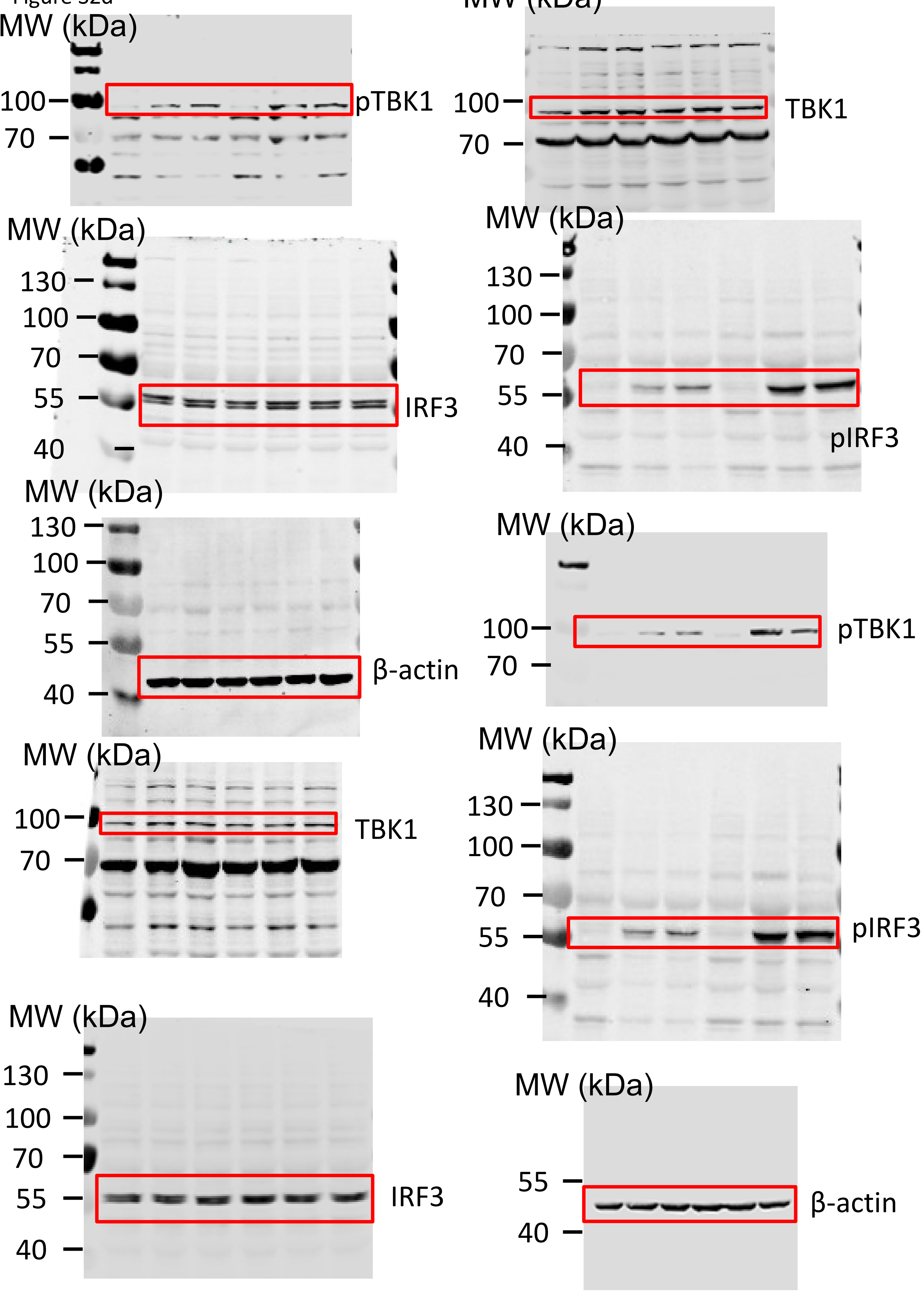
MW (kDa)
100
70
pTBK1
MW (kDa)
100
70
TBK1
MW (kDa)
130
100
70
55
40
IRF3
MW (kDa)
130
100
70
55
40
pIRF3
MW (kDa)
130
100
70
55
40
β-actin
MW (kDa)
100
70
pTBK1
MW (kDa)
100
70
TBK1
MW (kDa)
130
100
70
55
40
pIRF3
MW (kDa)
130
100
70
55
40
IRF3
MW (kDa)
55
40
β-actin

Figure S2g

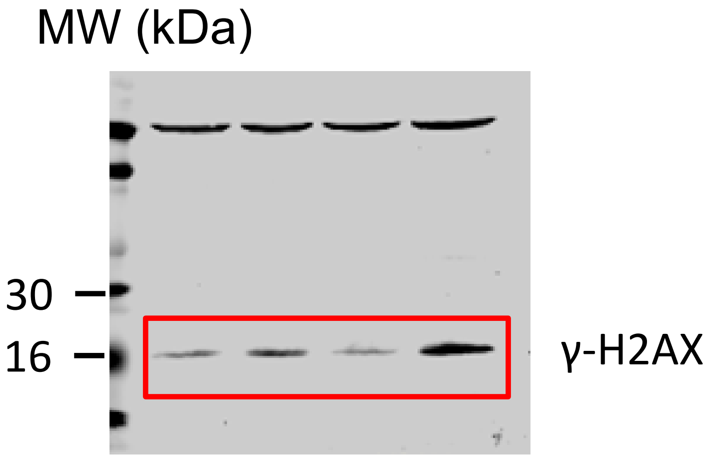

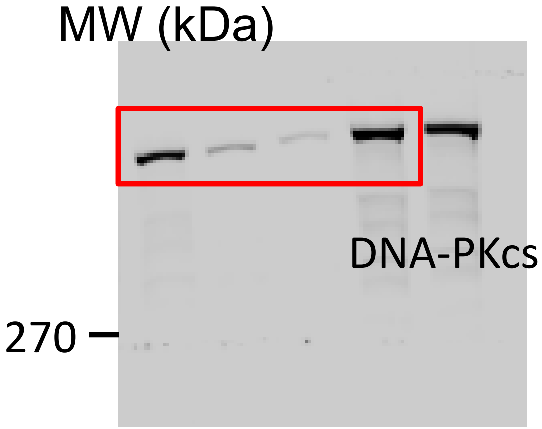

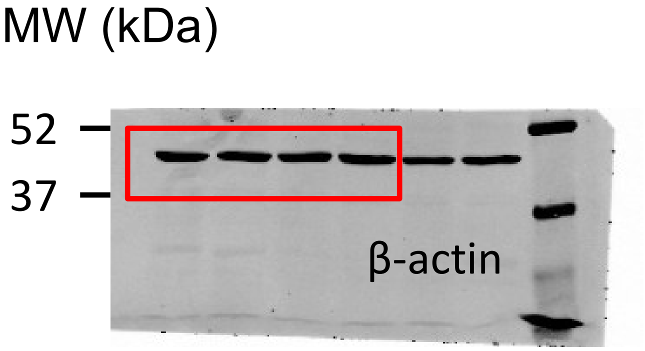

Figure 3a

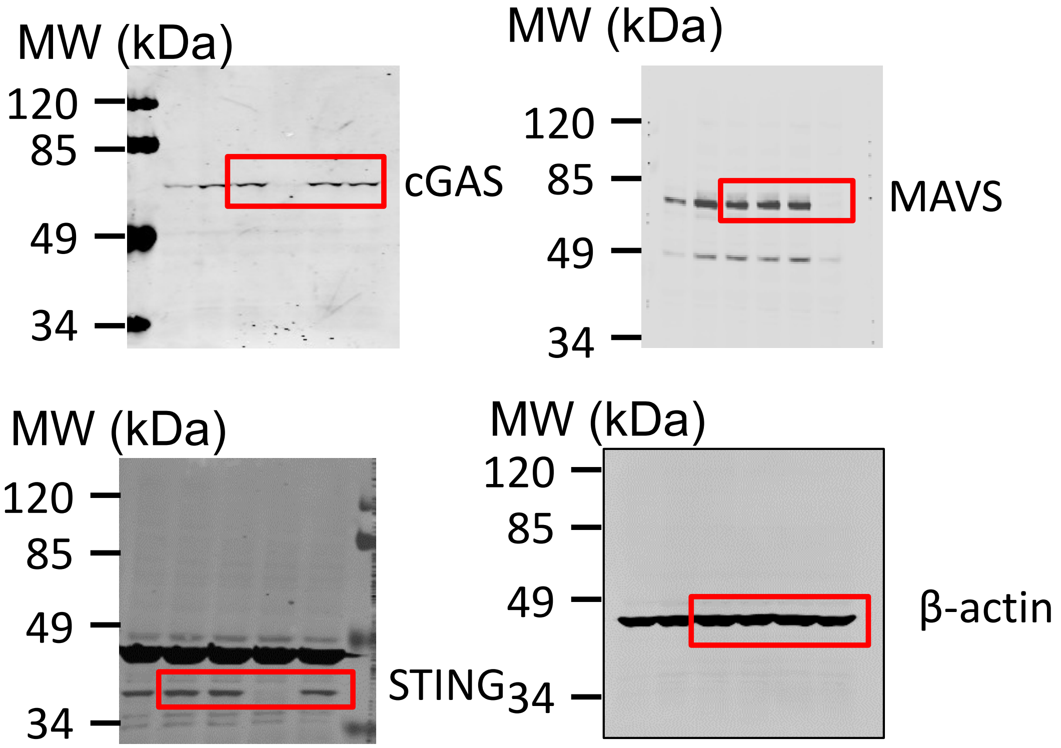

Figure 3d

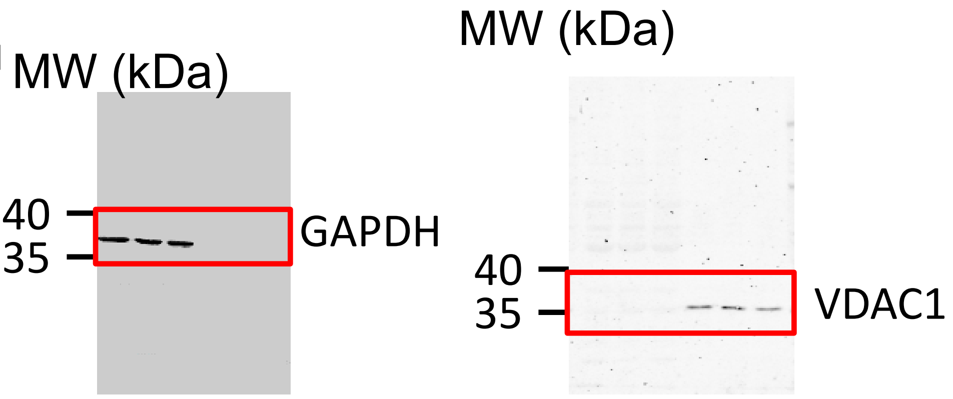

Figure 3f

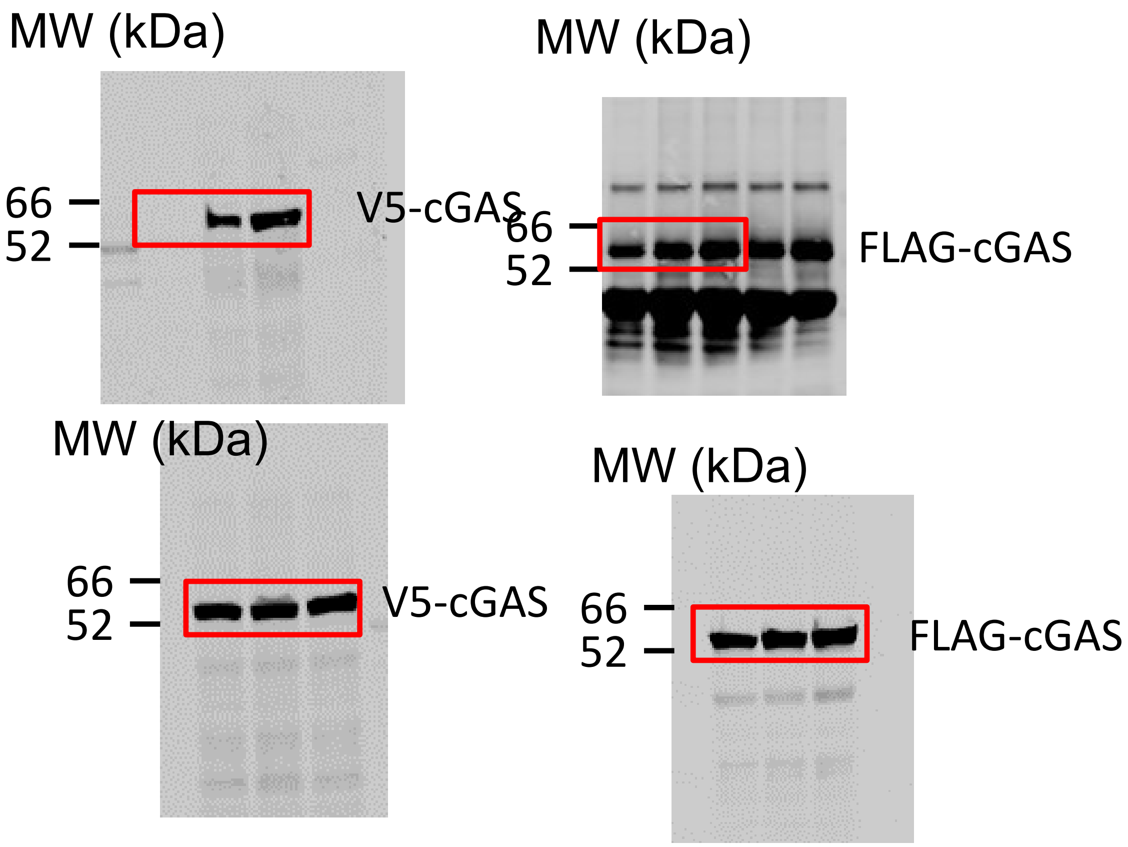

Figure S3e

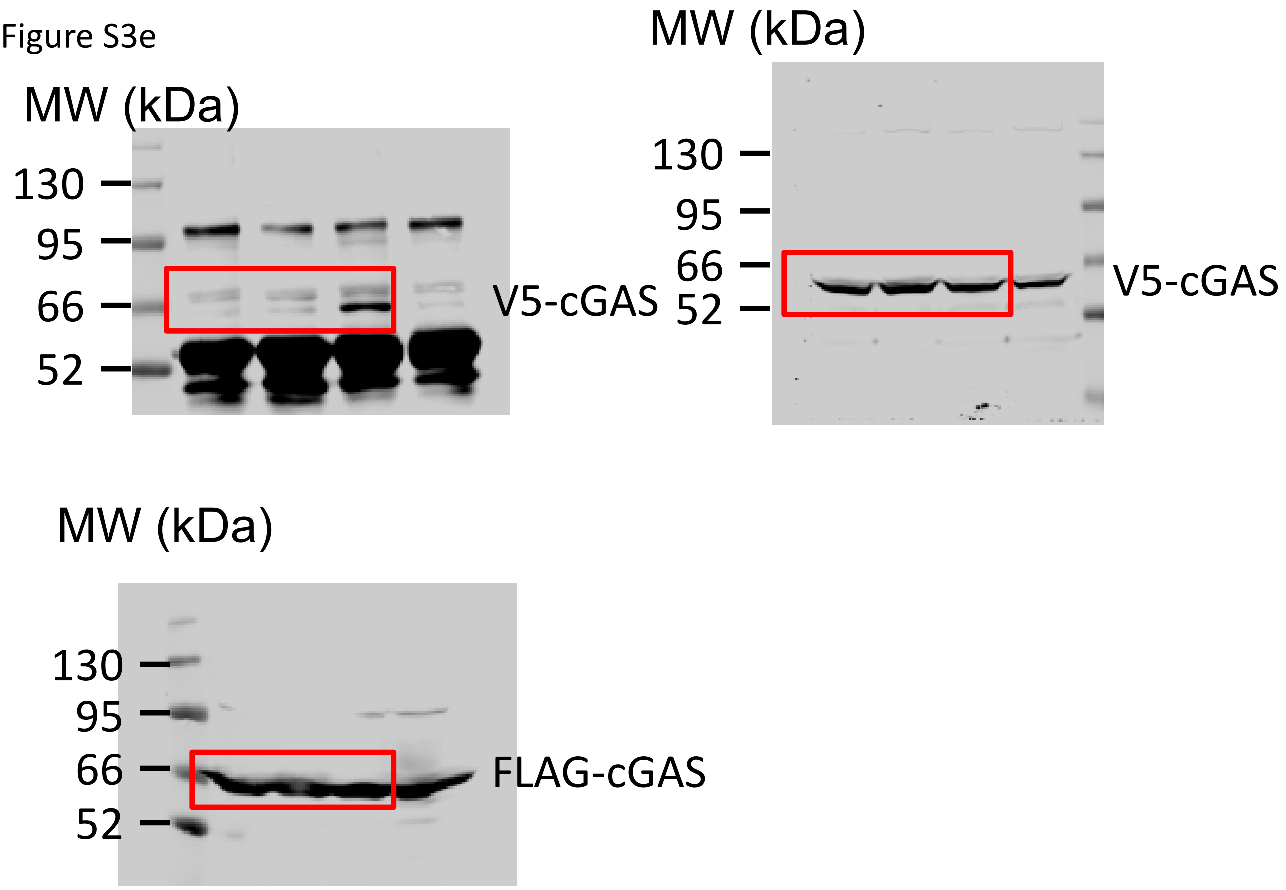

Figure 4a

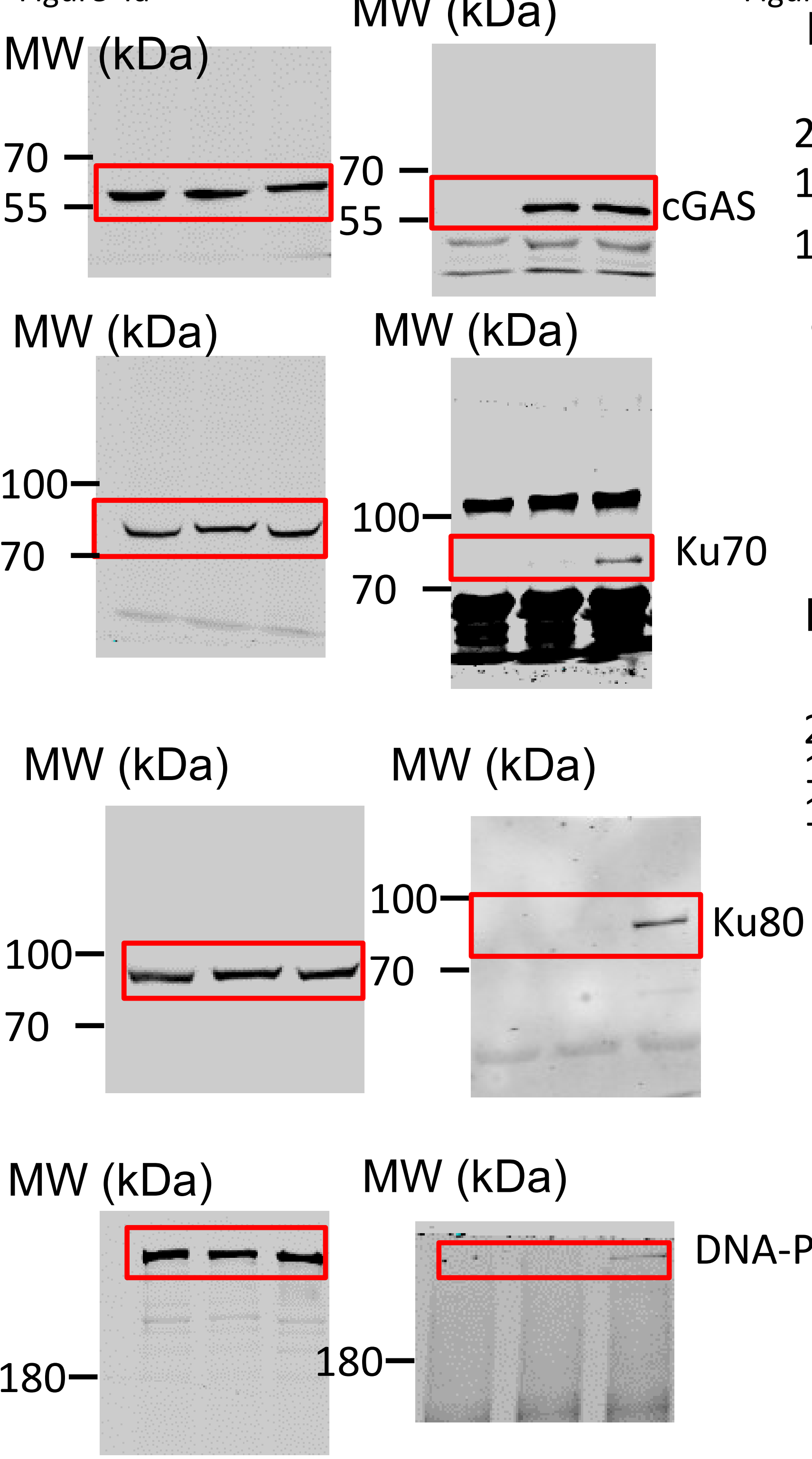

Figure 4b

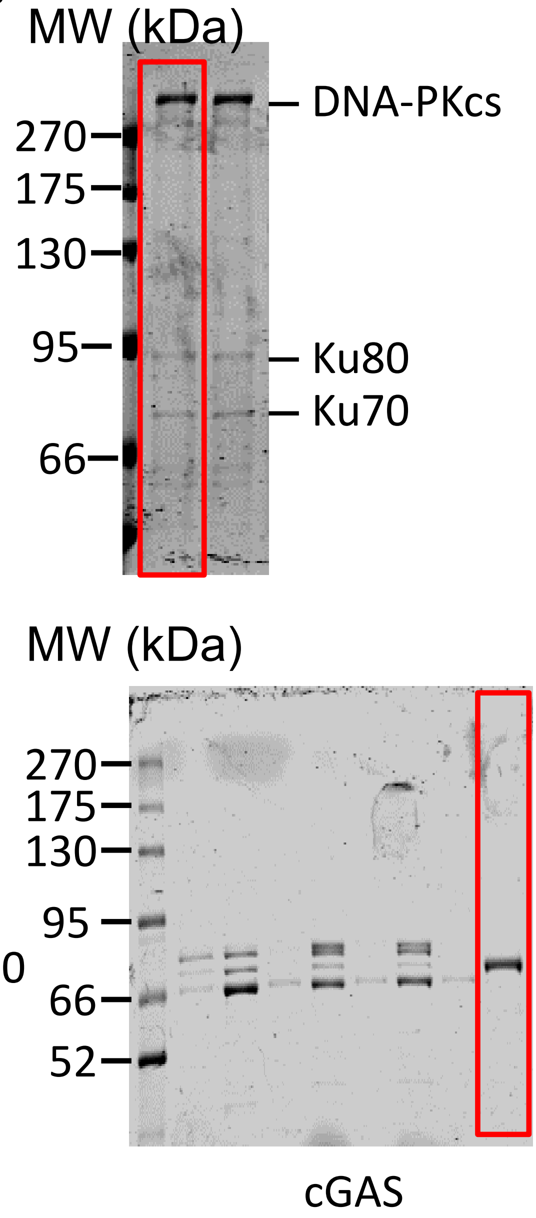

Figure 4c

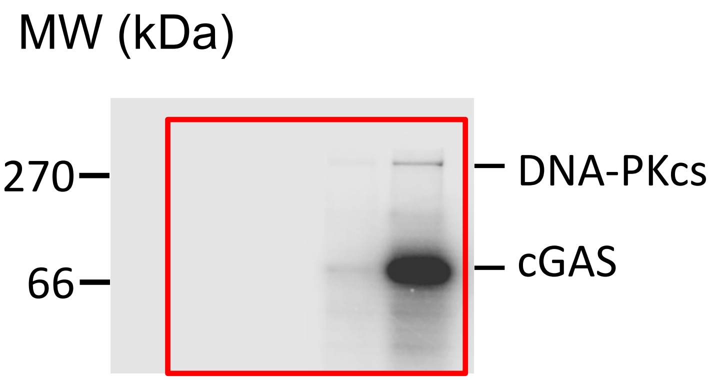

Figure 4d

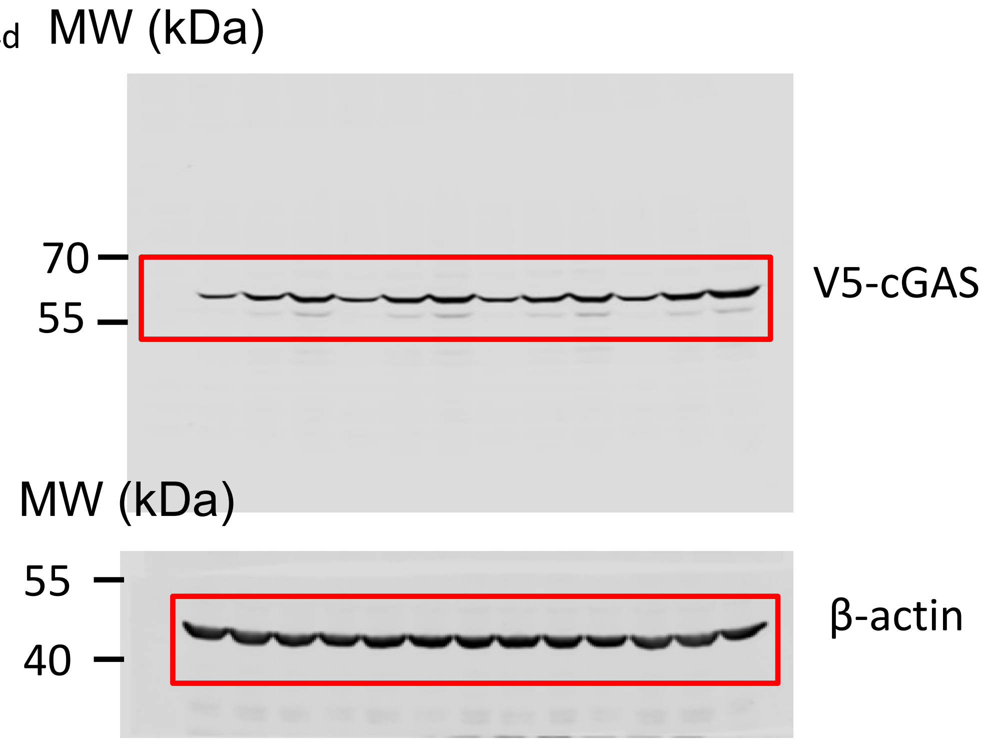

Figure 4e

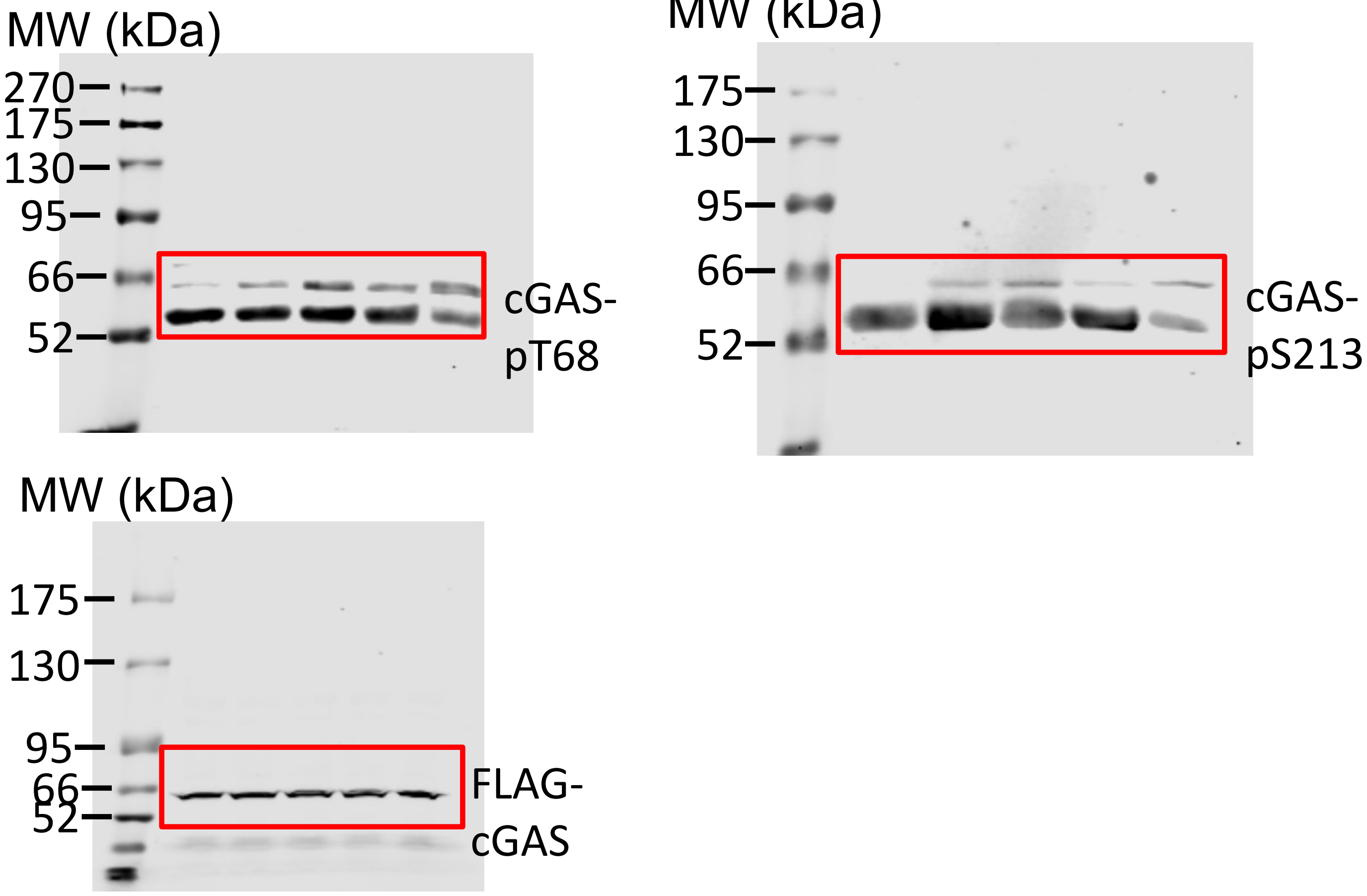

Figure 4f

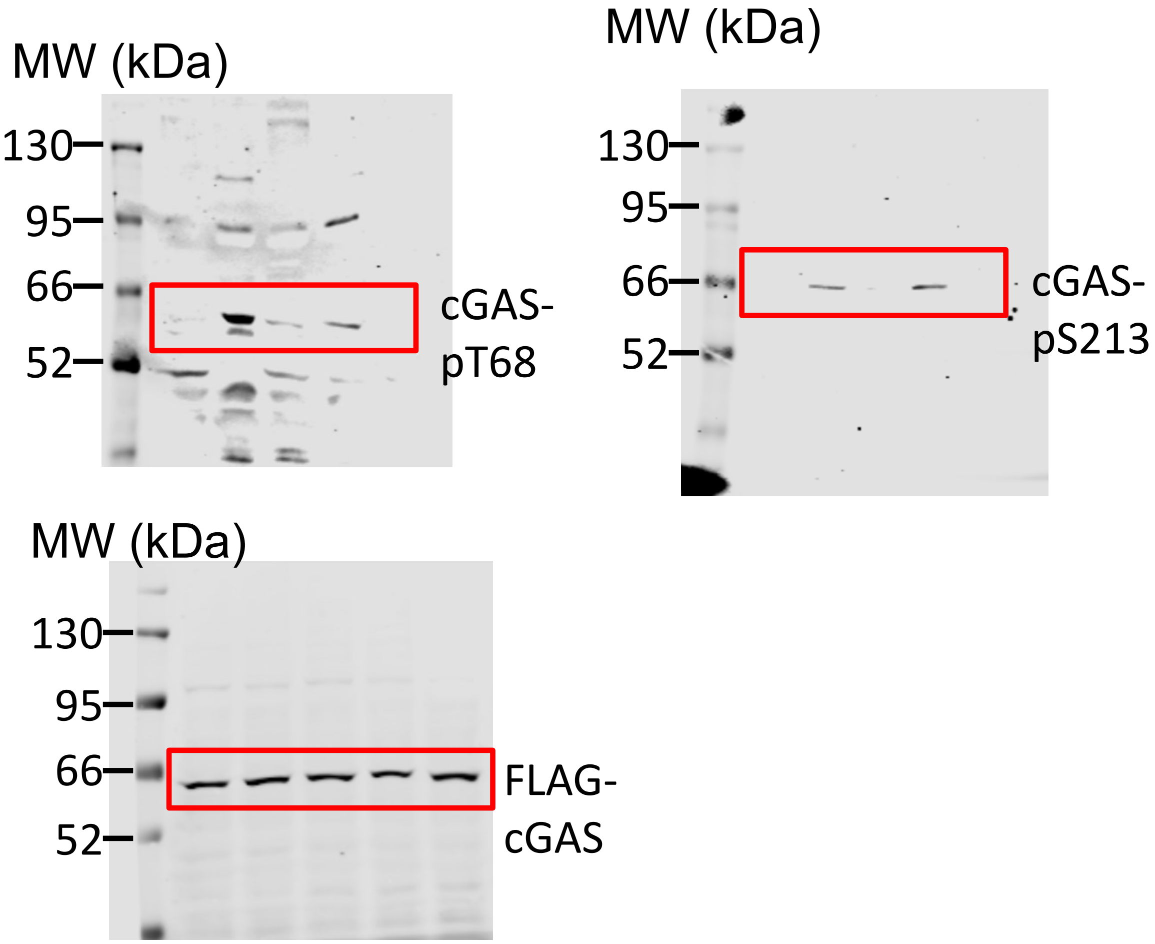

Figure S4a

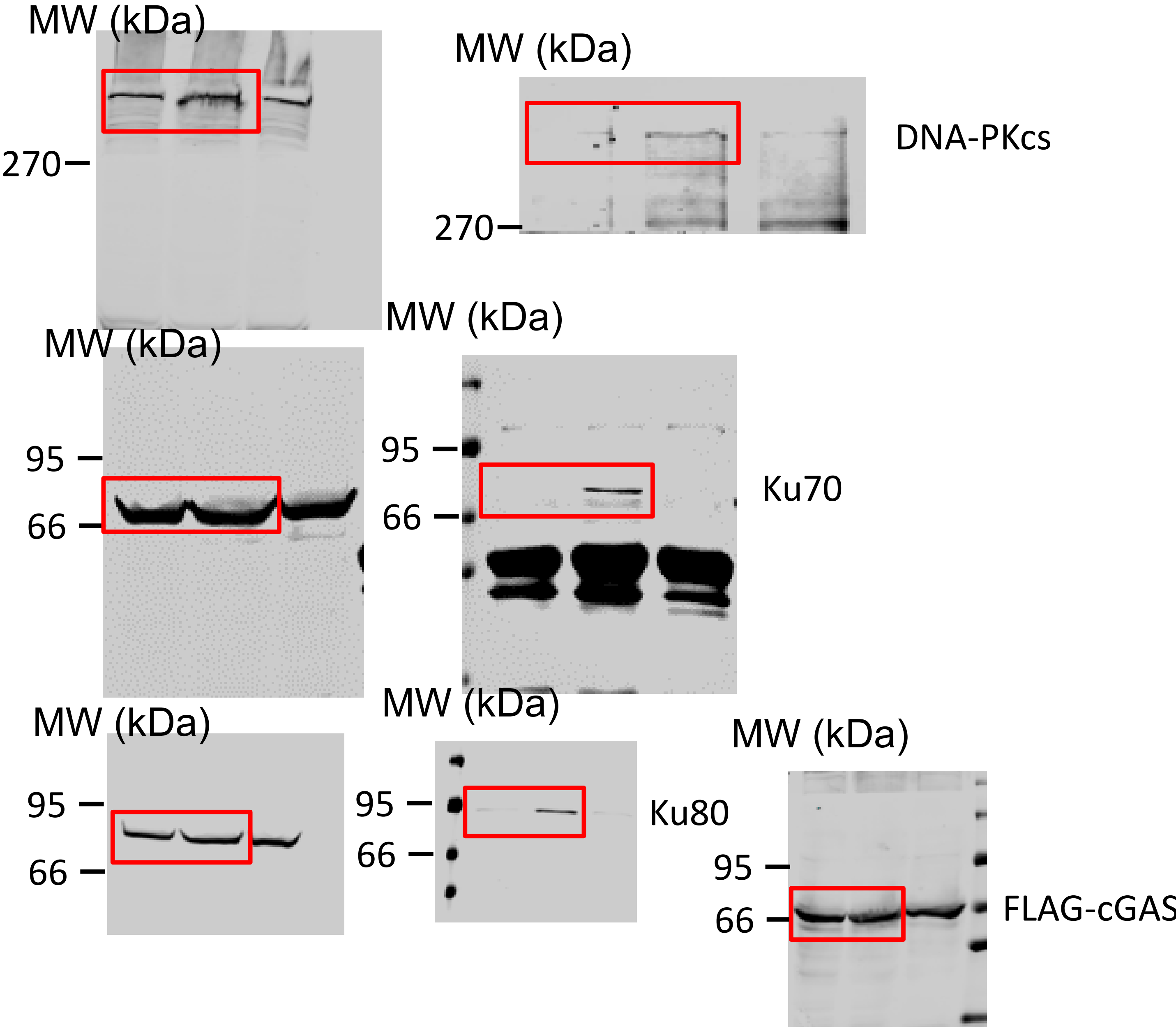

MW (kDa)
270
MW (kDa)
270
DNA-PKcs
MW (kDa)
95
66
MW (kDa)
95
66
Ku70
MW (kDa)
95
66
MW (kDa)
95
66
Ku80
MW (kDa)
95
66
FLAG-cGAS

Figure S4b

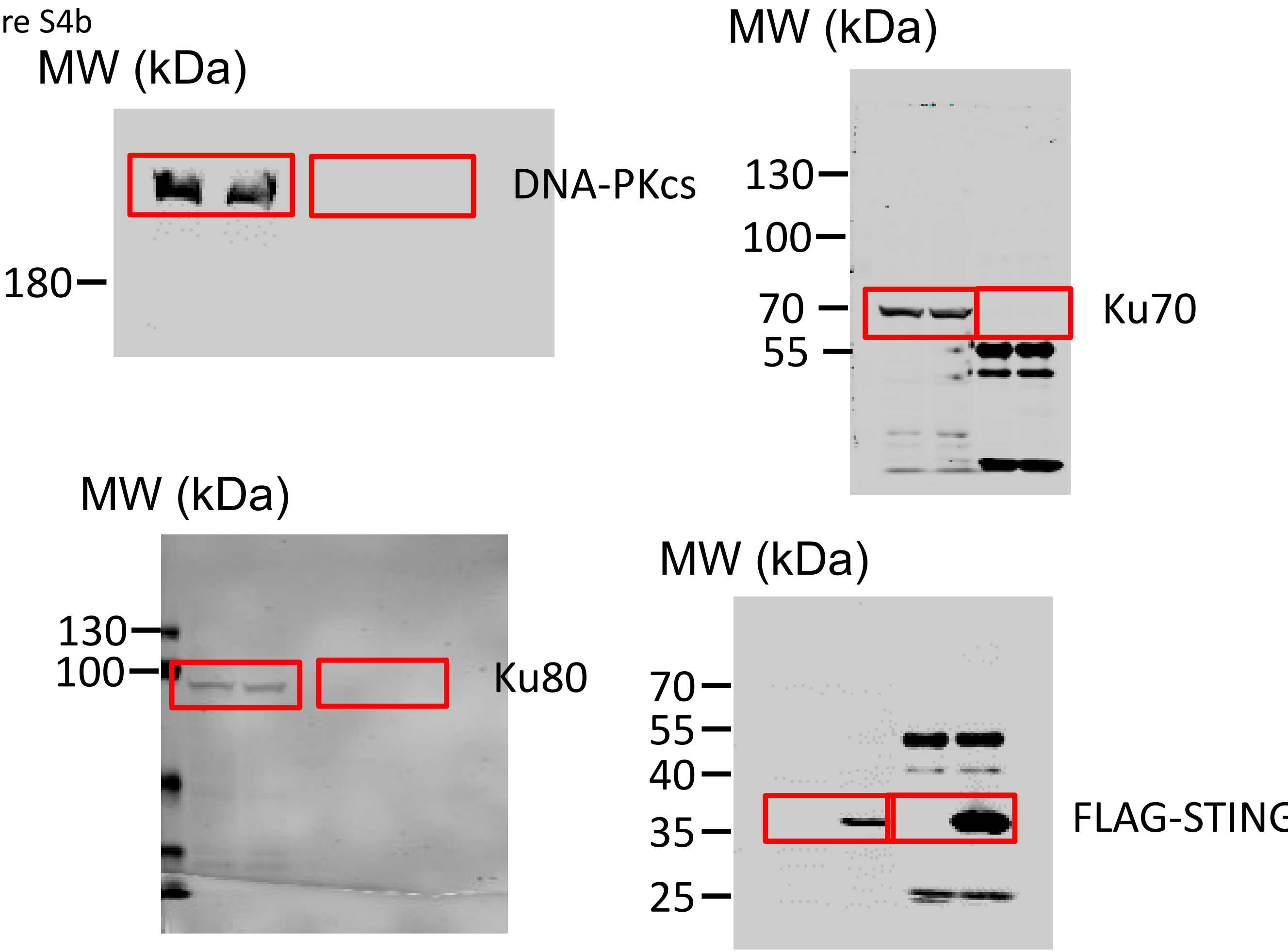
MW (kDa)
180
DNA-PKcs
MW (kDa)
130
100
70
55
Ku70
MW (kDa)
130
100
Ku80
MW (kDa)
70
55
40
35
25
FLAG-STING

Figure S4c

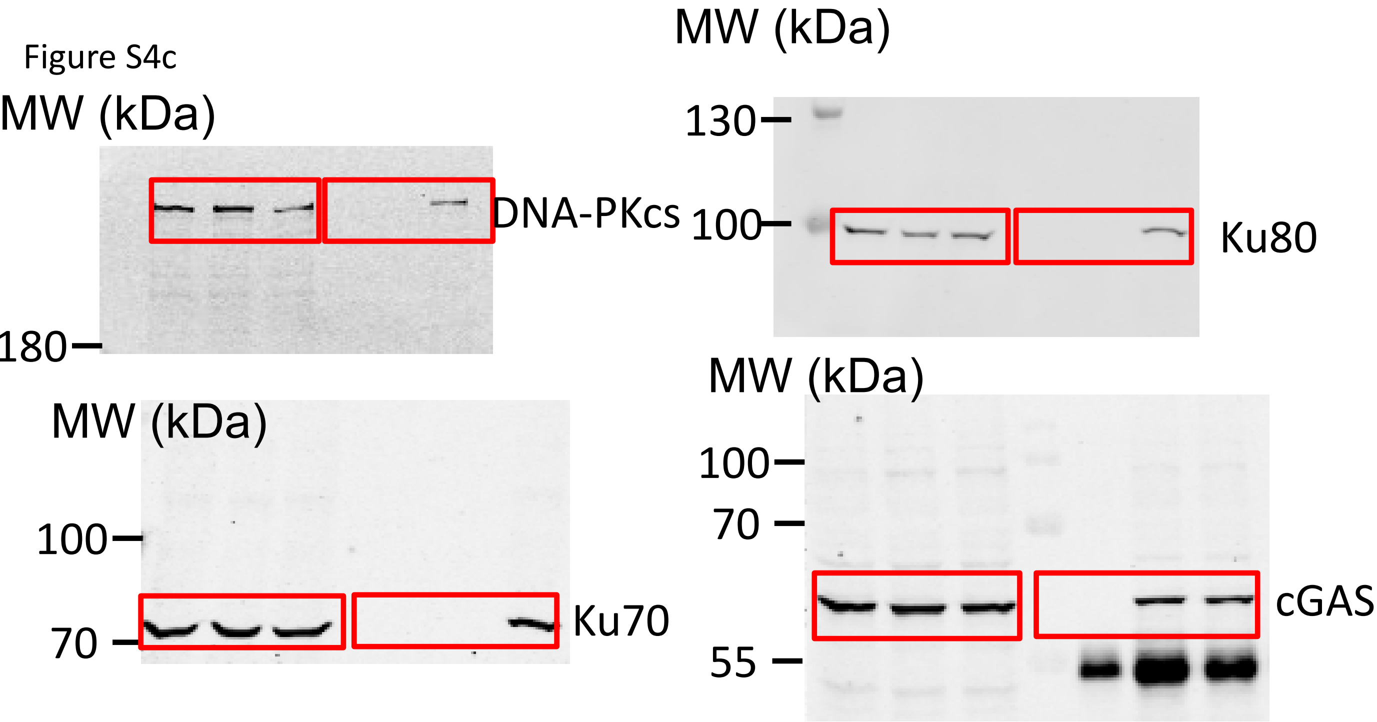

Figure S4e

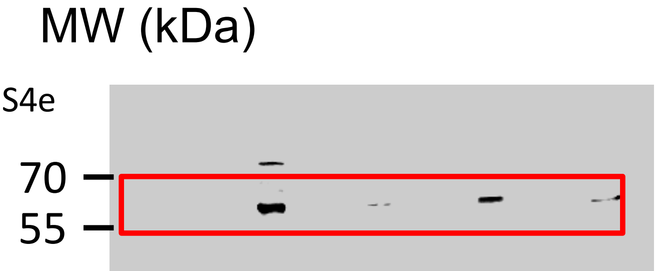

Figure S4f

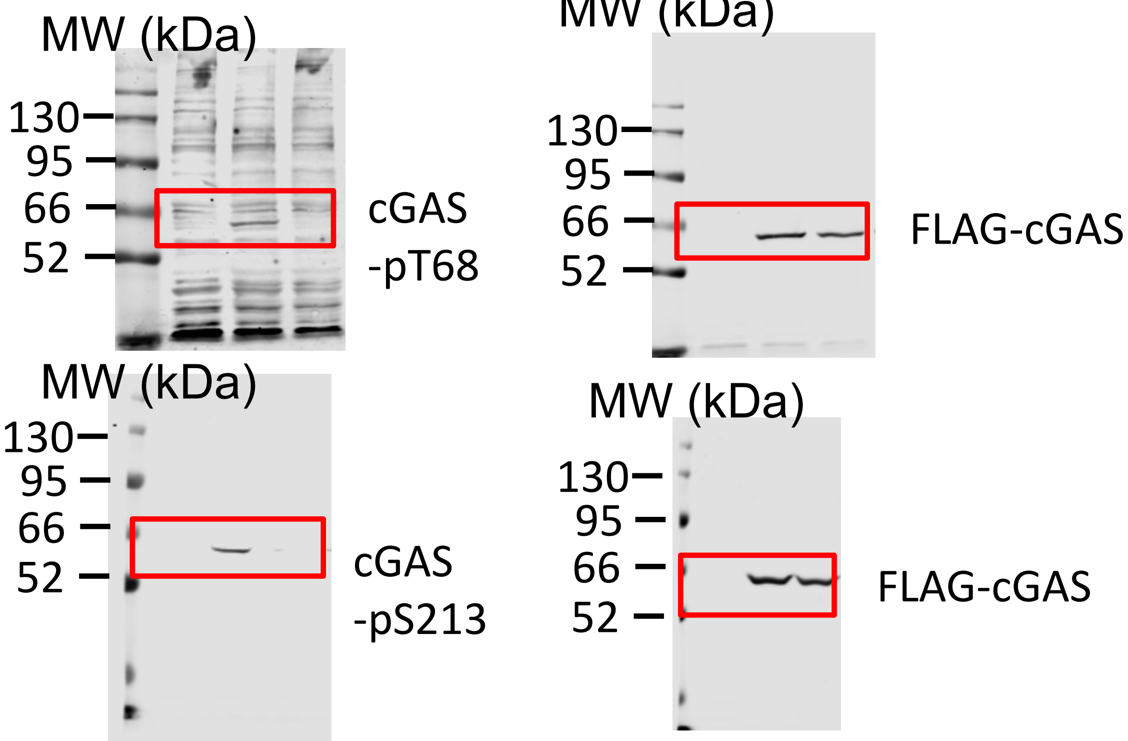

Figure S4g

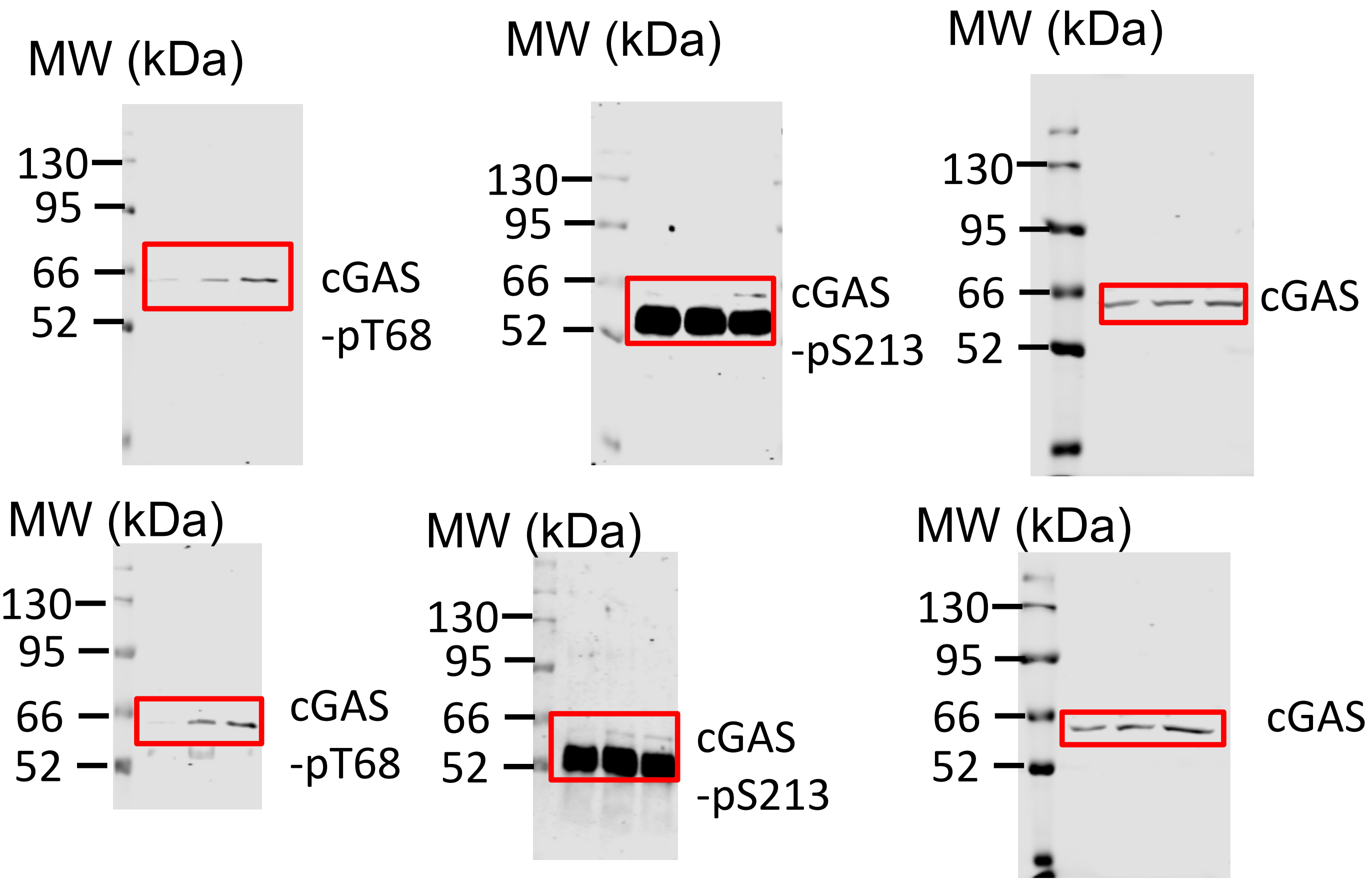
MW (kDa)
130
95
66
52
cGAS
-pT68
MW (kDa)
130
95
66
52
cGAS
-pS213
MW (kDa)
130
95
66
52
cGAS
MW (kDa)
130
95
66
52
cGAS
-pT68
MW (kDa)
130
95
66
52
cGAS
-pS213
MW (kDa)
130
95
66
52
cGAS

Figure 5a

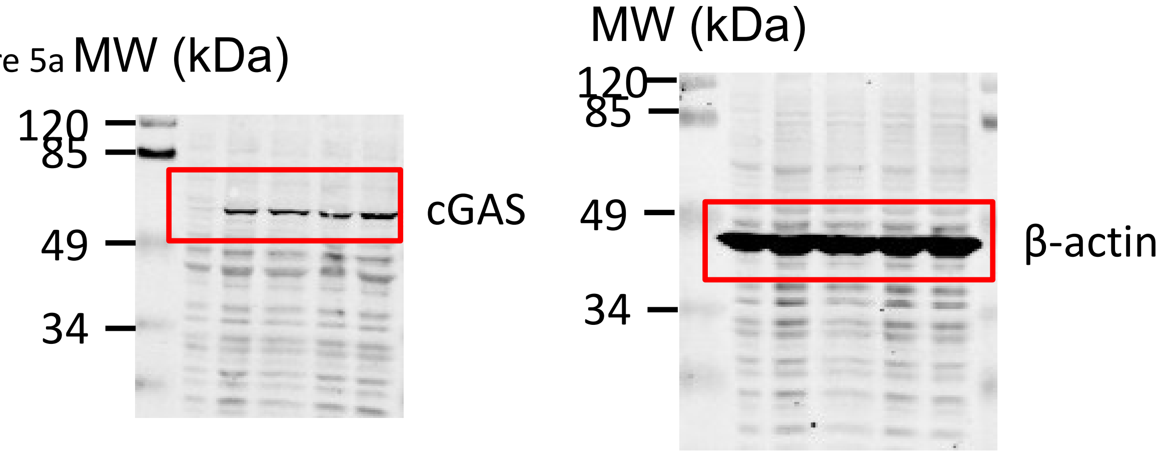

Figure 5d

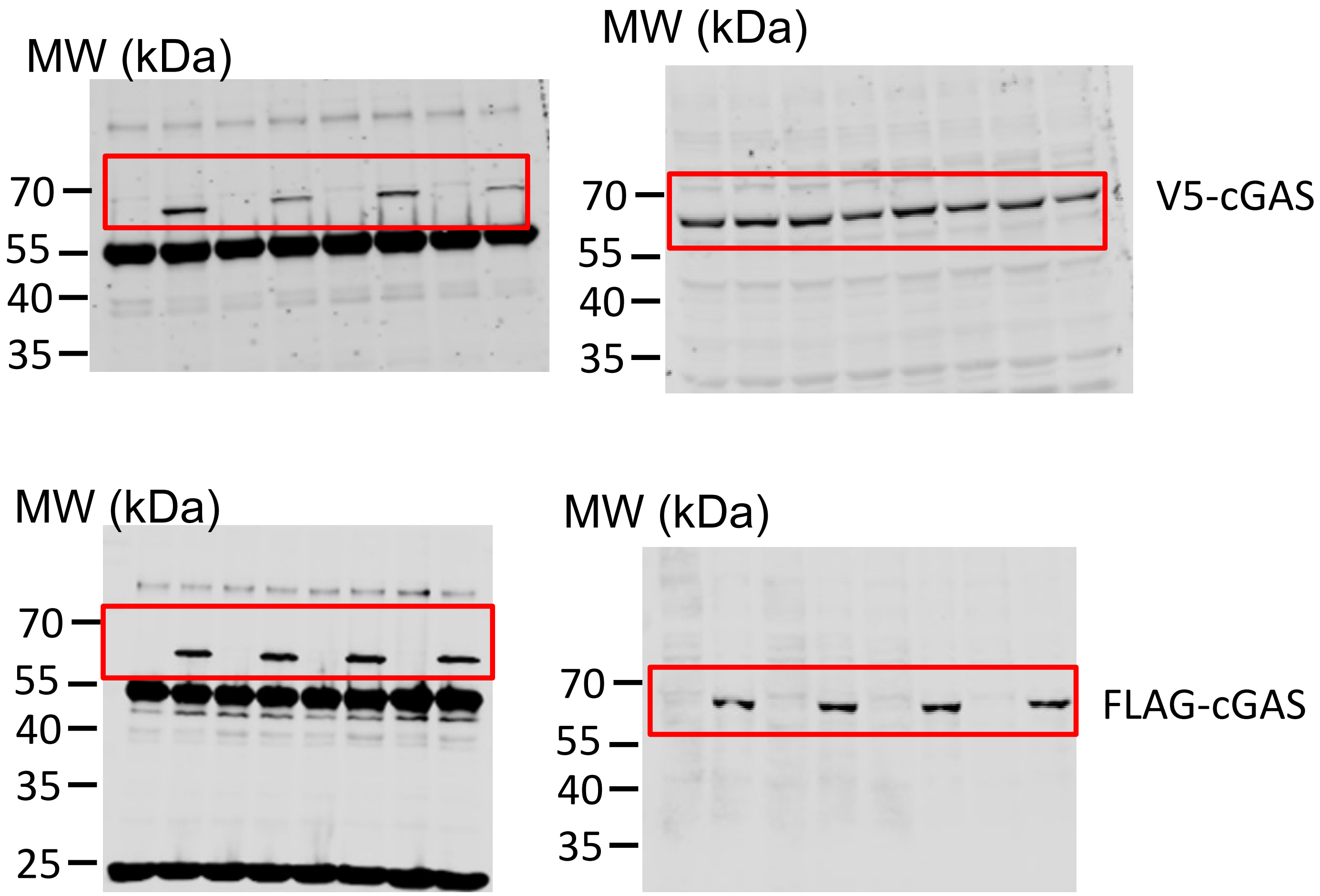

Figure 5e

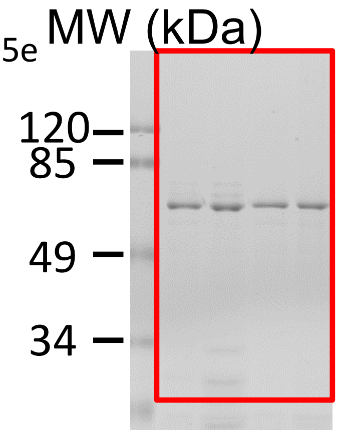

Figure 7d and Figure S7d

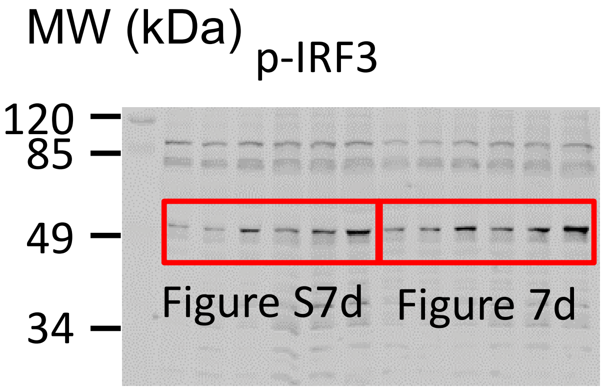

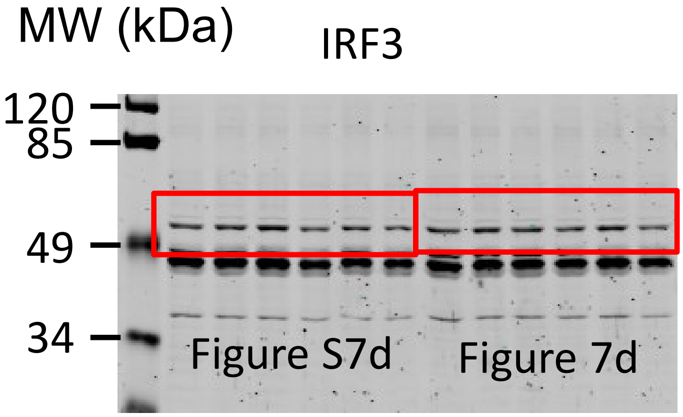

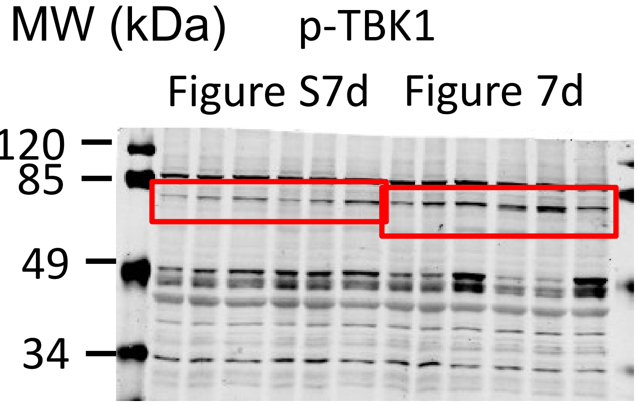

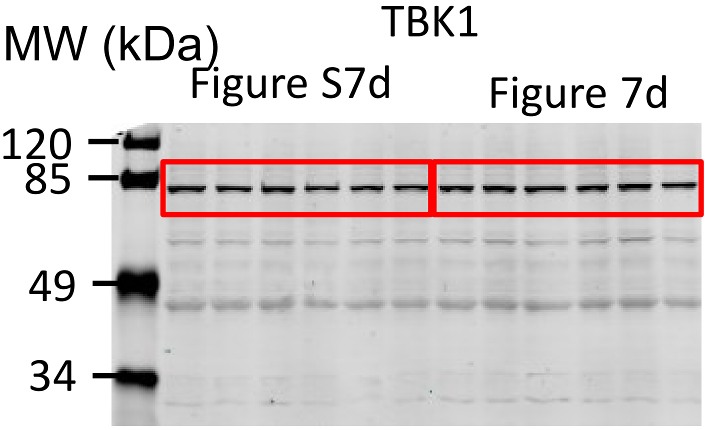

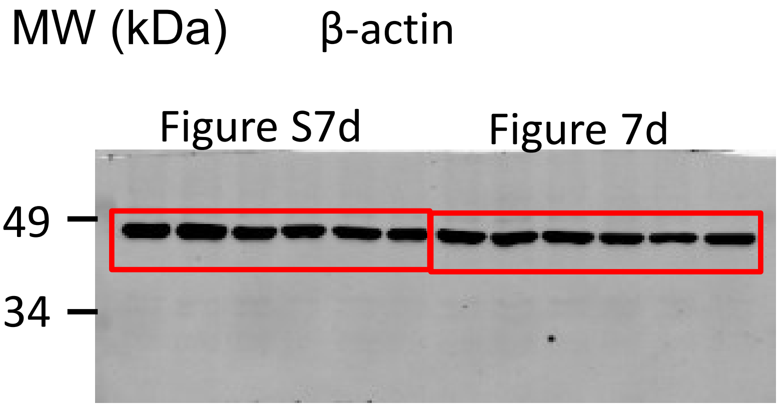

Supplement: Supplementary file 4 — Source Data [file 41467_2020_19941_MOESM4_ESM.zip › source data_uncropped gels.pdf]
